# Hydrophone placement yields high variability in detection of *Epinephelus striatus* calls at a spawning site

**DOI:** 10.1002/eap.70081

**Published:** 2025-08-06

**Authors:** Cameron J. Van Horn, Alli C. Candelmo, Scott A. Heppell, Croy R. M. McCoy, Christy V. Pattengill‐Semmens, Lynn Waterhouse, Laurent M. Cherubin, J. Christopher Taylor, William Michaels, James Locascio, Ali K. Ibrahim, Brice X. Semmens

**Affiliations:** ^1^ Scripps Institution of Oceanography University of California, San Diego La Jolla California USA; ^2^ Reef Environmental Education Foundation (REEF) Key Largo Florida USA; ^3^ Oregon State University Department of Fisheries, Wildlife, and Conservation Sciences Corvallis Oregon USA; ^4^ Department of Environment Cayman Islands Government George Town Grand Cayman, Cayman Islands; ^5^ U.S. Geological Survey, Minnesota Cooperative Fish and Wildlife Research Unit, Department of Fisheries, Wildlife, and Conservation Biology University of Minnesota St. Paul Minnesota USA; ^6^ Harbor Branch Oceanographic Institute Florida Atlantic University Fort Pierce Florida USA; ^7^ National Ocean Service, National Oceanic and Atmospheric Administration Beaufort North Carolina USA; ^8^ Mote Marine Laboratory Sarasota Florida USA

**Keywords:** automatic classifier, fish calls, FSA, hydroacoustics, movement ecology, spawning aggregation

## Abstract

Passive acoustic monitoring is a cost‐effective, minimally invasive technology commonly used to study behavior and population dynamics of soniferous fish species. To understand the strengths and limitations of acoustic monitoring for this purpose at fish spawning aggregations (FSA) requires an assessment of the variability in aggregation‐associated sounds (AAS) as a function of time, space, and proximity for spawning fishes of interest. Here, we evaluate temporal and spatial trends in the detection of AAS by Nassau Grouper (*Epinephelus striatus*) using an array of six hydrophones deployed across a large Nassau Grouper FSA at Little Cayman, Cayman Islands. We collected continuous data for nine days during a winter spawning season and subsequently used an automatic classifier to extract the embedded Nassau Grouper AAS. Using these data, we analyzed variability in spatiotemporal AAS detection rates across the array with a Bayesian mixed effects model. We found high variability in the detection of AAS across the spawning site, with positive correlations among neighboring hydrophone pairs trending toward negative correlations with distances exceeding 350 m. Indeed, temporal trends in AAS rates at the spawning site were approximately inverted at the two most distant hydrophones (~600 m). Across the hydrophone network, our model predicted strong positive effects of fish proximity, spawning behavior, and crepuscular periods on detected AAS. Our findings suggest hydrophone placement can strongly influence AAS detection rates and even basic temporal patterns in AAS across the spawning season. Given both the vagaries of movement and behavior of aggregating fish at spawning sites and the limits of AAS detection using standard monitoring tools, we suggest spawning site acoustic monitoring programs deploy hydrophone arrays of sufficient size to capture the site‐wide trends in AAS rates if possible; this is particularly true if researchers hope to compare/contrast AAS rates between spawning sites or across seasons for the purpose of population assessment.

## INTRODUCTION

Marine fisheries are globally important in the context of economic stability (US $164 billion in export value in 2018), societal function (260 million jobs), and food security (17% of total animal protein consumed globally), the latter of which is considerably important in tropical and subtropical coastal communities (FAO, 2020; Milich, 1999; Teh & Sumaila, 2013; Worm & Branch, 2012). Government actions to sustain fishery productivity, such as intensive fishery‐independent at‐sea surveys, are often more financially limited in these regions than those of industrialized nations. These fisheries thus operate in data‐poor conditions that threaten the ability to sustain catch over time. Because of this, there is a need to develop and employ cost‐effective monitoring tools that allow for informed management action. In these lower latitudes, many targeted fish species in need of effective monitoring methods are those that form highly dense and predictable fish spawning aggregations (FSAs).

These FSAs, or mass gatherings of fish for reproductive purposes, recruit individuals from potentially hundreds of kilometers away and occur over very short annual time scales (Domeier & Colin, 1997). Species that form FSAs presumably do so to maximize their reproductive fitness. However, because these events are often highly predictable in space and time, aggregating populations can be intensively fished once discovered, which in turn can lead to overexploitation and ultimately collapse of the fishery. More than half of all documented FSAs in the tropical West Atlantic no longer form (De Mitcheson et al., 2008). On the other hand, because FSAs concentrate the entire reproductive biomass of a region into relatively small areas at predictable times, they provide an opportunity to census stocks for the purposes of assessing status, recruitment, and recovery in depleted populations. To do so, however, requires monitoring tools that are effective, low cost, minimally invasive, and that can function in underwater environments that are otherwise difficult to access by researchers.

Passive acoustic monitoring (PAM), a widely used tool in bioacoustic research, is a rapidly growing technique for monitoring FSAs (Lindseth & Lobel, 2018). Commonly used to study terrestrial species such as birds, bats, and insects (Laiolo, 2010), PAM has been applied to a diverse range of marine soundscape projects (Gottesman et al., 2021; Lyon et al., 2019; Mann & Grothues, 2009). Acoustic monitoring lends itself to FSA monitoring because many species that form FSAs produce aggregation‐associated sounds (AAS) while aggregating (Rowell et al., 2017). Fish produce AAS to recognize conspecifics and signal mating readiness, thereby serving as a mechanism for sexual selection and reproduction (Lobel, 1992; Webb et al., 2008). Monitoring FSAs with deployable acoustic recorders (i.e., hydrophones) enables AAS data collection without the temporal or environmental (e.g., depth/light) restrictions that are often associated with visual fish survey methods (Luczkovich et al., 2008; Marques et al., 2013; Rountree et al., 2006). Furthermore, visual methods such as underwater visual census (UVC) or diver‐operated video (DOV), can be disruptive to fish behavior, and thus affect estimates of biodiversity and abundance (Emslie et al., 2018).

While PAM presents a minimally invasive strategy to monitoring FSAs, challenges remain, including (1) the need for time‐intensive classification of AAS from recordings, and (2) a poor understanding of the relationship between AAS call rates and the proximity/abundance of fish in relation to the hydrophone. The plethora of recorded acoustic data can pose a significant problem for timely analysis because of the inherent necessity to detect acoustic signatures, which is often done manually (Aalbers & Sepulveda, 2012; Rowell et al., 2012; Schärer et al., 2012). Advancements in the field of machine learning algorithms have produced novel solutions in the form of automatic classifiers (Munger et al., 2022; Stowell et al., 2019). Rapid automatic classification enables large swaths of raw acoustic data to be analyzed for specific spectral signatures in a fraction of the time and has been applied to fish species such as Atlantic cod (*Gadus morhua*) as well as several Sciaenids (Caiger et al., 2020; Monczak et al., 2019). For grouper species that form FSAs in the tropical Western Atlantic, Ibrahim et al. (2018, 2024) developed FADAR (Fish Acoustic Detection Algorithm Research), an automatic classifier designed to detect spectral signatures of AAS. The accuracy of data generated by FADAR and its variability across FSA sites is important to know when employing the software; despite this, the software is largely untested in the literature. We note here that while previous studies have often distinguished a subset of AAS that are putatively associated with courtship (courtship‐associated sounds; CAS), FADAR does not make this distinction, and thus our paper focuses on all call types at the aggregation (i.e., AAS).

Rapid automatic classification of AAS expands the potential for PAM; however, a poor understanding of how AAS detections vary in space and time persists. The accuracy of passive acoustic data relies on the successful transmission of an acoustic signal by a source to a recorder, the likelihood of which is high if the fish calls near the hydrophone. The spectral features of an AAS dictate its detectable range; however, this range is not likely to be known for a target species given the paucity of studies on sound‐producing fishes (Looby et al., 2022). Because passive acoustic methods often deploy a single hydrophone to monitor a population, fish movement during the FSA period can leave the hydrophone beyond the AAS detection range. While the high spatiotemporal predictability of FSAs may warrant dismissal of these concerns, observed shifts in the mass of spawning fish at several FSAs across years suggest reliance on past FSA geolocations can risk the accuracy of the data (Aguilar‐Perera, 2006; Caiger et al., 2020; Colin, 1992). Understanding the spatial and temporal variability of AAS detections is therefore important for PAM, though this has not been well explored to date. We attempt to resolve this knowledge gap by studying spatiotemporal trends in AAS production by a well‐studied species known to form FSAs, using an array of hydrophones placed across a known FSA site.

The Nassau Grouper (*Epinephelus striatus*) is a gonochoristic large‐bodied opportunistic predator whose commercial, cultural, and ecological value has been known throughout the Caribbean for generations (Colin, 1992; Domeier & Colin, 1997; Sadovy & Eklund, 1999; Smith, 1972). Nassau Grouper are a long‐lived, highly fecund, and late‐maturing species that form transient FSAs near shelf edges (Domeier & Colin, 1997; Sadovy & Eklund, 1999; Winemiller & Rose, 1992). Individuals demonstrate strong interannual fidelity to the FSA where they aggregate and reproduce, a behavior perhaps socially transmitted from older, more experienced fish (Blincow et al., 2020; Bolden, 2000; Dahlgren et al., 2016; Nemeth, 2012; Semmens et al., 2007; Starr et al., 2007). Migration to the FSA occurs around the winter full moons in the central Caribbean, where Nassau Grouper densities drastically increase and spawning behavior (stark coloration shifts and following, circling movements) is observed soon after (Archer et al., 2012; Colin, 1992; Sadovy & Eklund, 1999; Smith, 1972; Starr et al., 2007). Specifically, Nassau Grouper tend to release gametes within 30 min of the sun setting (Whaylen et al., 2004).

**FIGURE 1:**
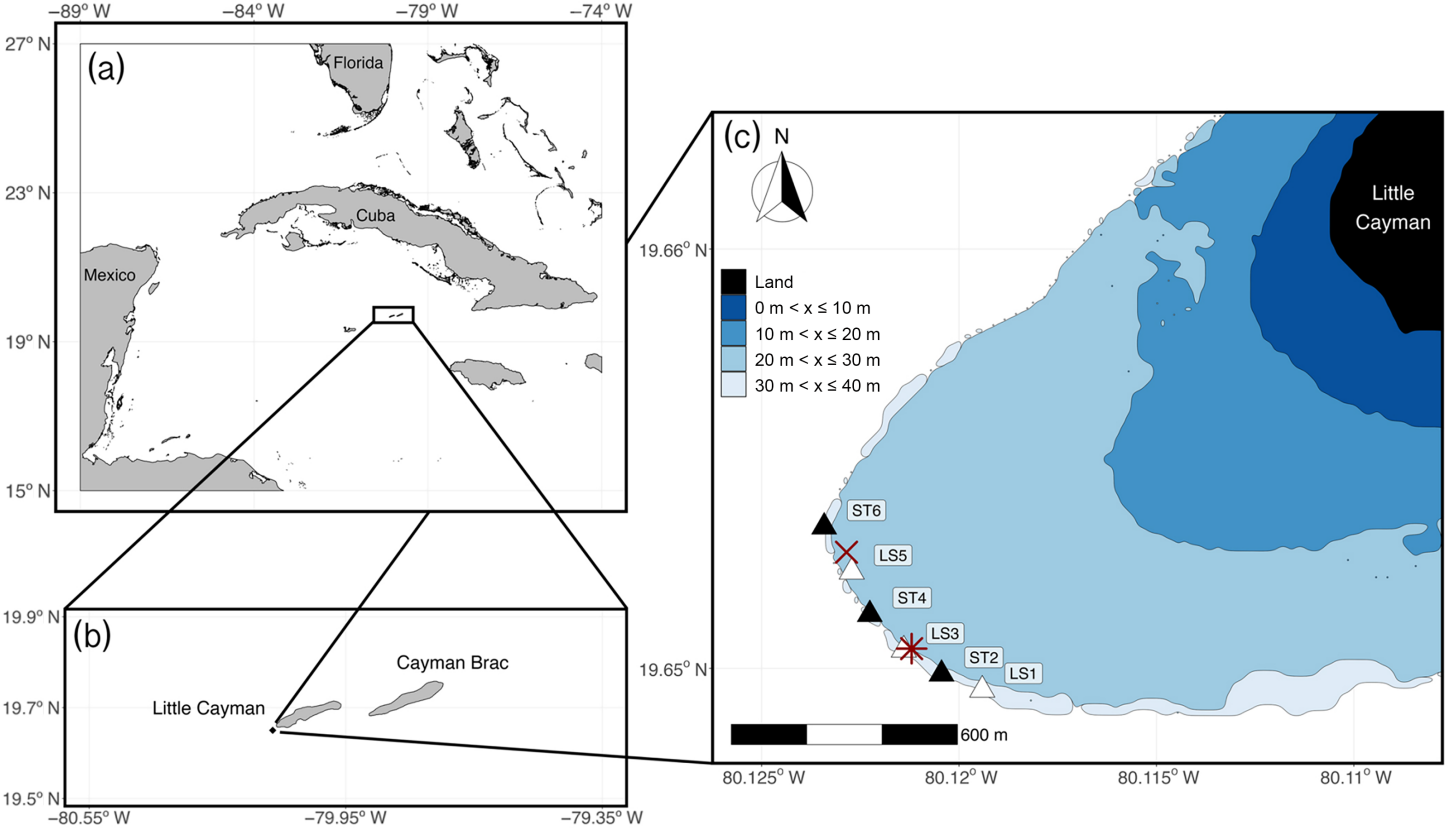
Little Cayman, Cayman Islands. (a) The Cayman Islands in the Caribbean Sea relative to Cuba, Florida, United States, and Mexico. Grand Cayman, the largest island in the Caymans, is just southwest of the drawn black box. (b) Little Cayman and Cayman Brac. A black dot off Little Cayman's west end signifies the fish spawning aggregation's (FSA) location. (c) Bathymetry of the west end of Little Cayman. Blues lighten to symbolize increasing depth on the order of 10 m, with land represented in black. Depth beyond 40 m is transparent. A dark red, bold “X” marks where divers first observed spawning individuals (between ST6 and LS5), and a dark red, bold “*” marks the traditional FSA location where fish gathered during the first few days of observation (adjacent to LS3). Triangles represent hydrophones and are labeled with their station. Black triangles signify SoundTrap (ST) hydrophones and white triangles represent LS1 (LS) hydrophones.

Nassau Grouper are listed as Critically Endangered by the International Union for the Conservation of Nature and Natural Resources due to overfishing at FSAs throughout its historical range (De Mitcheson et al., 2008; Sadovy et al., 2018; Sadovy & Eklund, 1999). Perhaps the most well documented collapse, and subsequent recovery, of the species occurred in the Cayman Islands, a small island nation to the southwest of Cuba (Figure 1a). In 1986, local fishers reported noticeable declines in Nassau Grouper catch, prompting the Cayman Islands Department of the Environment (CI‐DoE) to begin monitoring the known FSAs of the species. By 2001, ongoing heavy catch at the last remaining FSA of the species (off the west end of Little Cayman) motivated the Cayman Islands government to implement an Alternate Year Fishing Law in 2002 alongside other protective measures surrounding Nassau Grouper reproduction. By 2004, all known FSAs throughout the Caymans were protected by an 8‐year seasonal fishing ban at FSAs (Bush et al., 2006). This ban was subsequently made permanent (along with country‐wide seasonal non‐take rules and bag limits throughout the year), and continued efforts to conserve and monitor Nassau Grouper populations have proven fruitful, as recovery has been observed at FSAs off Little Cayman and Cayman Brac (Heppell et al., 2012; Stock et al., 2021; Waterhouse et al., 2020).

Here we present a case study on spatiotemporal distributions of Nassau Grouper AAS across the west end Little Cayman FSA site during the winter spawning season in 2020. The acoustic, behavioral, and reproductive ecology of Nassau Grouper at this FSA site is part of an ongoing monitoring program (The Grouper Moon Project; GMP), and thus lends itself to an intensive acoustic survey under known conditions of fish presence, spatial distribution, and reproductive behaviors. Furthermore, as this FSA is thought to host one of the largest known remaining aggregations of Nassau Grouper, behavior at this location, including AAS production, likely represents the best approximation of what courtship behaviors might look like under “natural” Nassau Grouper FSA conditions.

Using an array of six simultaneously recording hydrophones, we recorded the FSA soundscape over a 9‐day spawning period, and then parsed the recordings through the FADAR AAS classifier. We subsequently evaluated the performance of FADAR relative to manual classification methods using standard accuracy metrics to ensure generated data reflect the true soundscape at each hydrophone. With the resulting AAS detections, we compare spatial distributions of average hourly AAS detection rates through qualitative and quantitative methods to determine the spatial synchrony/asynchrony in AAS patterns at locations across the FSA area. Lastly, we assess drivers of variability in AAS by using a Bayesian mixed effects model of Nassau Grouper AAS detections as a function of variables presumed to affect calling behavior (e.g., time of day, day of spawning season) as well as the proximity of aggregating fish to the hydrophones. Collectively, these analyses provide an assessment of the spatial, temporal, and biological factors that influence hydrophone‐specific AAS detection rates.

## METHODS

### Study site

A large Nassau Grouper FSA (approximately 5000 fish in 2018; Waterhouse et al., 2020) occurs off the west end of Little Cayman (Figure 1b,c) and has been the target of numerous projects studying the ecology and recovery of Nassau Grouper populations (e.g., Heppell et al., 2009, 2012; Stock et al., 2021; Waterhouse et al., 2020; Whaylen et al., 2004, 2007). Much of the research in this region (including this paper) has been conducted at the west end Little Cayman FSA site by GMP, a collaborative group of researchers from the CI‐DoE, Reef Environmental Education Foundation (REEF), and academic partners. Grouper Moon has had a profound impact on the recovery of the species, as demonstrated by recent findings of stock recovery in some local populations (Waterhouse et al., 2020). As of 2016, conservation measures by the Cayman Islands government include: no‐take zones covering 45% of the total shelf area of the Cayman Islands, seasonal closures on all Nassau Grouper harvest and possession from December 1 to April 30, and strict take and gear restrictions in open season (Waterhouse et al., 2020). For a detailed description of the west end Little Cayman FSA site, see Kobara and Heyman (2008) and Whaylen et al. (2004).

### Data collection

#### Hydrophone array deployment

To monitor Nassau Grouper AAS across the spawning site, we deployed a six‐hydrophone linear array along the western shelf edge of Little Cayman from February 8 to February 17, 2020 (Figure 1c). This time period generally reflects the average residence time of Nassau Grouper at aggregation sites (Archer et al., 2012; Starr et al., 2007), with the full moon observed on February 9, 2020. Divers stationed the hydrophones in a linear path along the shelf edge at roughly 30 m depth with a 150 m distance between each (estimated by divers towing a 150‐m line between deployments). At a subset of hydrophone stations, divers deployed surface marker buoys, and a surface support team used a Garmin Etrex 20x GPS to geolocate the stations. We subsequently estimated the coordinates of the remaining stations using GPS waypoints taken at drop points of the diver teams and known distances between stations.

We used three Loggerhead Instruments LS1 (Loggerhead Instruments, Inc.) acoustic recorders (LS) and three Ocean Instruments SoundTrap Model 300HF (ST; Ocean Instruments, Auckland, New Zealand), and alternated placement of these models at each station along our array (Figure 1c). STs recorded continuously at a sample rate of 48 kHz with high preamp gain (which results in a sensitivity of −172 dB) and no high pass filter. LSs recorded continuously at a sample rate of 192 kHz and a sensitivity of −170 dB with no high pass filter. Due to the resource‐limited nature of this fishery, the hydrophones we used for this study were shared by other researchers conducting studies in the region, and thus their programming was preset, rather than intentionally chosen for this study. Audio was captured onto 256‐GB memory cards as 16‐bit .wav files of 6‐h and 1‐min increments for STs and LSs, respectively, with date and time upon recording embedded within each file name. We stationed the hydrophones between 09:00 and 10:00 on February 8 and retrieved them the morning of February 17 between 09:00 and 10:00 (these and all times listed throughout are in Eastern Standard Time, the local time of the Cayman Islands).

#### GoPro camera deployments

**FIGURE 2:**
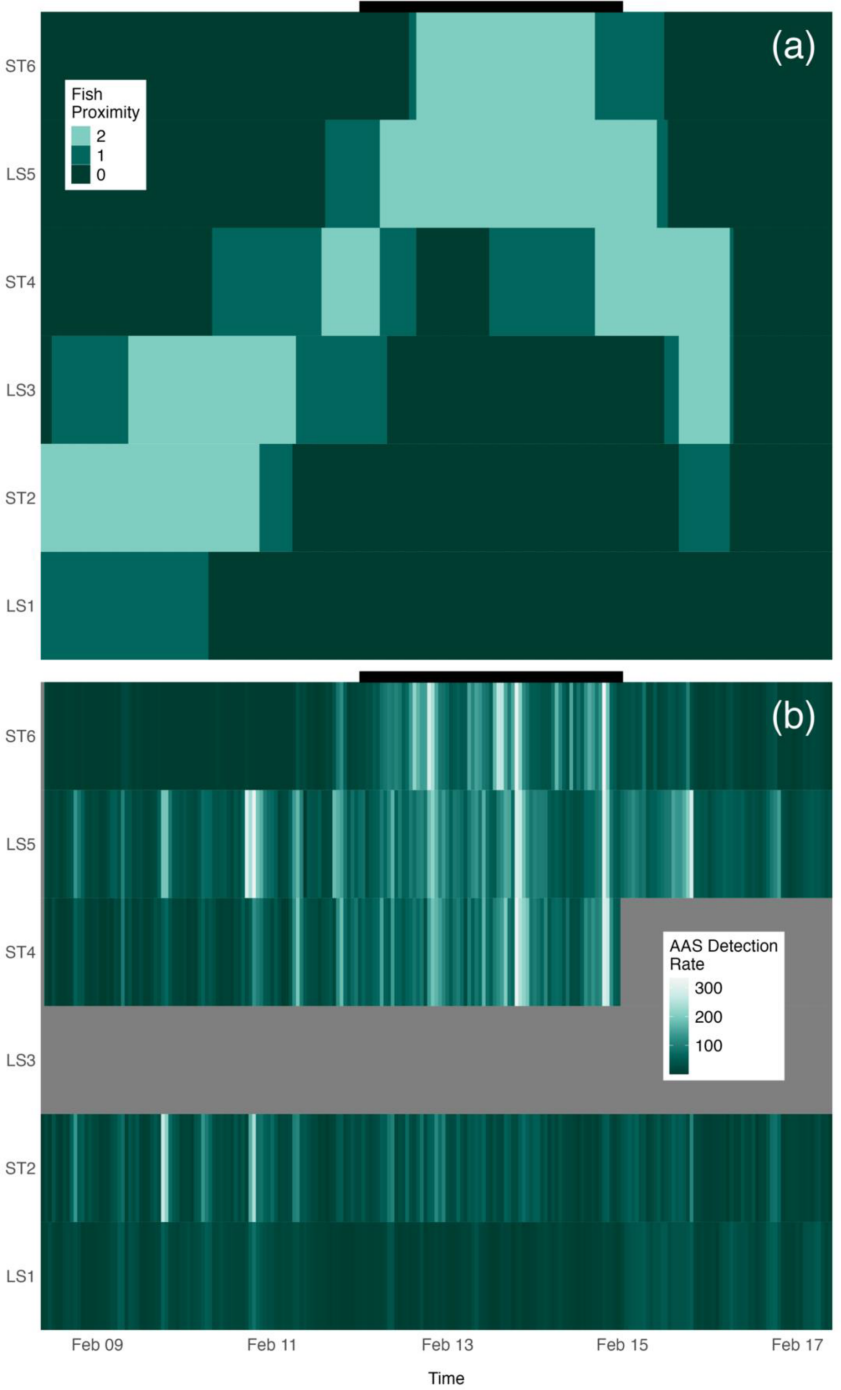
Heat maps of (a) categorized proximities of the bulk of Nassau Grouper (*Epinephelus striatus*) to each hydrophone per hour (see *Data analysis* for category descriptions) compared to (b) effort normalized aggregation‐associated sounds (AAS) detection rates at each hydrophone per hour. (a) Categorized proximities of the bulk of Nassau Grouper to each hydrophone per hour. As dark green panels lighten, the bulk of Nassau Grouper approach the hydrophone, with the lightest green signifying within 20 m of the hydrophone. (b) Continuous effort normalized AAS detection rates at each hydrophone. Dark greens lighten to white to signify increasing rates of AAS detected. Gray regions indicate when no data were collected. Black bars positioned above both panel (a) and (b) signify days when divers observed Nassau Grouper spawning (February 12 0:00 to February 15 0:00).

To estimate Nassau Grouper presence and movement around the FSA area, divers stationed GoPro HERO3 White cameras opportunistically along the array throughout the study period (LS1 omitted). To extend battery life, we installed CamDo TL‐004 intervalometers into the extra battery slot of the GoPros and programmed them to turn on and record for 2 min every 18 min. Beginning at ST2, divers opportunistically retrieved and redeployed the GoPros multiple times each day, repositioning the cameras nearest to where they observed the bulk of Nassau Grouper. Because divers never observed the bulk of Nassau Grouper near LS1, they did not deploy GoPros at that station (Figure 2). Six GoPros were used in total with no more than two at a given station. Divers installed the GoPros within several meters of a given hydrophone on the reef bed with nonuniform angle positioning (i.e., some faced parallel to the floor while others faced upward around a 45° incline). In instances where a station had two GoPros, the cameras were positioned on different parts of the adjacent reef and angled opposite each other for a better composite image.

#### Video transects of the FSA

We opportunistically collected video transects within the FSA area when fish schooled into a band along the shelf edge. To collect a video transect, a diver used a Canon 1DX Mark II—Sigma 18–35 mm f/1.8 Lens (Canon U.S.A., Inc.)—Nauticam Wide Angle Conversion Port (WACP; Nauticam International Ltd.) and a Dive X Piranha P1 Scooter (Dive Xtras, Inc.) to traverse the length of the band while keeping aggregating fish within the camera frame. Divers collected video transects during multiple dives throughout the spawning period. We examined each video census to determine (1) the daily/hourly locations of the band of fish at the spawning site, and (2) where the fish were most abundant during each dive using underwater visible landmarks and marking buoys.

#### In situ diver observations

Researchers conducted three dives each day in the morning, afternoon, and evening with exceptions during the evening of February 8 (the first day of data collection), the afternoon of February 16, and the afternoon and evening of February 17 (the final two days of data collection). Divers noted fish location, presence, abundance, and movement around the spawning site. Divers also visually confirmed Nassau Groupers spawning as part of a separate long‐term research project that required the collection of eggs from spawning events in situ. For spatial accuracy, divers approximated fish position and movement using established moorings, landmarks, and stations along the shelf edge (see Appendix S1: Figure S1a,b for detailed map). These in situ diver observations, in concert with GoPro recordings and video transects, were later used to approximate the location for the bulk of the aggregating fish, as well as estimate northern and southern boundary limits of the FSA.

### Data processing

#### Call classification

The spectral qualities and statistics of Nassau Grouper AAS are well known (Rowell et al., 2018; Schärer et al., 2012; Wilson et al., 2020). Because of the volume of acoustic data generated in our study, we used the automatic AAS classifier FADAR3 (Ibrahim et al., 2018). Nassau Grouper produce three unique identified AAS that we included in this study. (1) In agonistic encounters, Nassau Grouper often produce a sequence of individual pulse sounds (termed pulse trains). These pulse trains are often produced when Nassau Grouper exhibit distressed or alarmed behavior regardless of season; however, they are consistently recorded at FSAs during spawning seasons (Fish & Mowbray, 1970; Moulton, 1958; Wilson et al., 2020). The individual pulse segments last on the order of 0.09 ± 0.02 s (±1 SD) while the full train can last up to 3 s and has a peak frequency of 77.4 ± 30.3 Hz (Schärer et al., 2012; Wilson et al., 2020). (2) During courtship, Nassau Grouper emit a low‐frequency tonal sound that lasts 1.6 ± 0.3 s and has a peak frequency of 99.0 ± 33.6 Hz (Schärer et al., 2012). (3) Lastly, Rowell et al. (2018) identified a third Nassau Grouper AAS described as akin to that of a heartbeat. The AAS has properties similar to the pulse train, with a mean duration of 0.37 s and mean peak frequency of 117.7 Hz (Rowell et al., 2018). FADAR does not specify which Nassau AAS it detects, thus the AAS counts in this study are nonspecific to a call type. Wilson et al. (2020) hypothesized a fourth AAS but we did not include it in this study as FADAR had not been trained to detect it at the time of data generation.

FADAR operates by processing an acoustic signal and (1) denoising the signal through discrete wavelet transformation, (2) processing the signal through long short‐term memory (LSTM) layered networks, and (3) attributing discriminative features of the signal for final classification of the origin species. FADAR divides the time series of the acoustic spectra into signals of 2‐s lengths. Each of these 2‐s bins can only have 1 attributable AAS, if any, regardless of species. The classifier sums AAS detections and links their associated 2‐s bins within each audio file (e.g., an AAS detected at seconds 33–37 will be noted as 1 AAS occurring between seconds 32 and 38).

#### Hydrophone array

To analyze spatial variability of Nassau Grouper AAS during the recording period, the large volume of data generated by PAM required a standardized effort to produce AAS detection rates per hour for each hydrophone. We began this process by considering differences in data generation by each hydrophone model and instances of anthropogenic sources. We segmented the acoustic data from ST hydrophones, initially recorded in 6‐h lengths, into 1‐min intervals to facilitate temporal comparisons with the automatically segmented LS data.

Boats frequented the aggregation site during recording, creating low‐frequency interference at sporadic intervals. While daily research dive operations constituted most of this boat traffic, small local fishing vessels periodically visited the site in the morning while fishing for tuna just off the shelf. The frequency range of vessel noise (often between 0 and 600 Hz) overlaps with Nassau Grouper AAS and masks detections while present. Also, we found that FADAR confused some instances of low‐frequency noise (possibly from boat traffic) with Nassau Grouper calls and overestimated AAS counts therein. For these reasons, we removed all segmented acoustic data containing vessel noise from our analysis. Because we recorded over 1000 h of the FSA soundscape, the likelihood of encountering anthropogenic noise in any given minute was low. To efficiently identify instances of vessel noise interference across the array, in each dataset, we subset 1 min for every 5 recorded minutes. We then processed the subset recordings into Audacity 2.4.2 to visually inspect the spectrograms for anthropogenic noise. If at least half of the file's length (30 s) contained anthropogenic noise between frequencies of 0 and 600 Hz, we labeled it as having mechanical (non‐biological in origin) interference. Because we did not inspect every minute recorded, we assumed identified instances of interference also occurred 2 min prior to and after the observed instance. We used a 2‐min window because, in the event we observed two consecutive minutes of mechanical noise in our subset (e.g., at 10:00 and 10:05), the total time between these points would be noted as containing mechanical noise. In total, 2722 min contained interference, or roughly 4.4% of all acoustic data. No dataset from any hydrophone contained interference in more than 8.2% of its data (LS5). Further, select hours in the morning, afternoon, and evening at each hydrophone station contained more minutes of interference at each hydrophone station (see Appendix S1: Figure S2) which coincides with diving activity described in Data collection. After removing these files, we tracked minutes annotated within all hours at each hydrophone to normalize AAS detections per hour by annotation effort.

#### GoPro and diver observations

We sought to categorize the proximity of the main mass of Nassau Grouper to each hydrophone for all recorded daylight hours. We inspected all GoPro files to categorize Nassau Grouper abundances per hour into one of five categories: absent (0), individual (1), few (2–10 individuals; 2), many (11–100; 3), and abundant (>100; 4). Because individuals would swim in and out of the camera's view, we categorized abundances via MaxN counts (Whitmarsh et al., 2017). We then combined these GoPro categorized abundances with the estimated position and relative abundances from diver observations and video transects to create a holistic view of the proximities of the bulk of Nassau Groupers to each hydrophone for all observable hours. We divided these composite proximities into three categories: not observed (0), where neither video transects, diver observations, nor GoPros observed fish within 100 m of the hydrophone; nearby (1), where GoPros, diver observations, or video transects observed the bulk of Nassau Groupers within 100 m of the hydrophone; and present (2), where GoPros, diver observations, or video transects observed the bulk of Nassau Groupers within 20 m of the hydrophone.

### Data analysis

#### Confusion matrices

To assess FADAR's automatic classification performance, we developed confusion matrices and calculated relevant estimates of accuracy indices. First, we randomly sampled 200 min of recordings from each hydrophone for analysis in FADAR (post removal of anthropogenic interferences). We then had a trained researcher listen to the acoustic samples in Audacity to identify instances of Nassau Grouper AAS. To prevent any bias in our evaluation of FADAR, we enumerated AAS along the same constraints as that of the classifier. Specifically, FADAR assigns time stamps to detected AAS by dividing the acoustic time series into bins of 2‐s lengths and assigning no more than 1 AAS to each bin. This method may prevent FADAR from, for instance, detecting a short tonal AAS that occurs within the same 2‐s bin as a proximal pulse AAS. We therefore required the trained researcher doing manual AAS identification to provide time stamps in intervals of 2‐s for all identified AAS and barred any given AAS from temporally overlapping with another AAS. To be specific, in the event multiple AAS were present in a given 2‐s interval, the researcher retained the earliest temporal call in the 2‐s window. We termed detections heard by the human researcher as “true” AAS and FADAR as “predicted” AAS. The comparison of these two metrics forms confusion matrices: true positives (agreed presence), true negatives (agreed absence), false positives (predicted presence that was absent) and false negatives (predicted absence that was present; Table 1). FADAR predicts if AAS are present in 2‐s windows, thus in each minute (the length of an analyzed file) the classifier makes 30 predictions. Because AAS presence is a relatively rare event, many of these predictions are likely to be true negatives, adding positive bias in accuracy estimates of FADAR. Therefore, we defined true negatives as full minutes with no detected or present AAS. When we sampled 200 min from each hydrophone, the subsets from LS1 and ST6 yielded substantially fewer AAS detections (regardless of method) relative to ST2, ST4, and LS5. We therefore added additional random draws to the test datasets of these hydrophones to meet a minimum threshold of 100 “true” AAS present per hydrophone in the test data. Confusion matrix data for each hydrophone, split by FADAR and the human researcher when applicable, are available in Table 1.

**TABLE 1:** Data from evaluating classification of Nassau Grouper (*Epinephelus striatus*) aggregation‐associated sounds (AAS) by automatic (FADAR, Fish Acoustic Detection Algorithm Research) and manual (human) efforts within all hydrophone datasets.

Station	Classifier	Sample size	*n*	TP	TN	FP	FN	Act Pres	Act Abs	Pred Pres	Pred Abs
LS1	FADAR	294	317	64	215	1	37	101	216	65	252
ST2	FADAR	200	185	15	76	0	94	109	76	15	170
	HUMAN	200	271	131	96	17	27	158	113	148	123
ST4	FADAR	200	275	5	161	0	109	114	161	5	270
	HUMAN	200	309	173	87	9	40	213	96	182	127
LS5	FADAR	200	337	204	80	10	48	252	90	214	128
ST6	FADAR	235	307	90	170	3	44	134	173	93	214

*Note*: The malfunction of LS3 prevented data collection; thus, it had no data to present. Because ST4 stopped recording near midnight on February 15, FADAR's performance within ST4 contains bias toward data collected during the first seven days. Only ST2 and ST4 required human evaluation due to poor performance by FADAR within their respective datasets.

Abbreviations: Act Abs, actual absence; Act Pres, actual presence; FN, false negative; FP, false positive; Pred Abs, predicted absence; Pred Pres, predicted presence; TN, true negative; TP, true positive.

We calculated the following statistics for all confusion matrices: accuracy, misclassification rate, rates for all true/false positive/negative terms, and Cohen's Kappa. Accuracy is a simplistic metric that quantifies the rate at which the classifier is correct (with misclassification rate as the inverse), while the true/false positive/negative terms provide nuance (e.g., if the classifier is more accurate among positive or negative agreement). However, as described prior, the presence of AAS in a given minute of data is somewhat rare. There is thus considerable random chance for a classifier to correctly predict the absence of AAS and artificially have high accuracy as a result. The Cohen's Kappa metric accounts for this by measuring the classifier's true performance against how well it would perform under random chance and assigning a score between −1 and +1 (McHugh, 2012). Variables to inform all evaluative statistics included the four true/false positive/negative terms along with summed values for predicted positives, predicted negatives, actual positives, actual negatives, and total detections (present and absent).

Due to FADAR's poor performance on some of our hydrophones (likely due to hardware malfunctioning; see below), we conducted a manual (human) classification effort on data from these hydrophones. Because these datasets contained nearly 26,000 cumulative minutes, we subset 1 min for every 5 min of acoustic data (identical to that of the anthropogenic parsing effort in 2.3). We then loaded the acoustic .wav files into Audacity to visualize the acoustic spectra for AAS identification and restricted observable frequencies between 0 and 600 Hz, with 20‐dB gain and a range of 80 dB. Within Audacity, we used a frequency algorithm with a Hamming window size of 16,384 and a zero‐padding factor of 1. To maintain consistency among AAS extraction in all hydrophones, our manual classification mimicked FADAR's execution by enumerating AAS solely through visual identification in spectrograms. Like FADAR, our manual classification detected AAS to 2‐s windows with no more than one AAS detection per window.

Using the above data products, we evaluated the performance of manual classification effort identically to that of FADAR. We defined visually detected AAS as “predicted” and audial AAS detections as “true.”

#### Model

We constructed a Bayesian hierarchical modeling framework to assess the drivers of spatiotemporal variability in Nassau Grouper AAS production. For it to run, we prepared the data by summing Nassau Grouper AAS detections per hour at each hydrophone for all hours observed by all hydrophones in the study period. We also removed sporadic hours that contained no observable minutes of AAS.

Because we are interested in the spatiotemporal variability of AAS, we used the rate of detected AAS for hydrophone *j* at hour (*R*) of the study (*V*_*j,i*_) as a Poisson distributed response variable. To account for the nonuniform observed minutes at each hydrophone for each hour, we separated the AAS detection rate term into the log of AAS detections per hydrophone and hour (μ_*j,i*_) and the log of minutes observed per hour at each hydrophone (τ_*j,i*_). This latter exposure term represents the variability in observable minutes per hour at each hydrophone that ultimately affects a hydrophone's ability to detect all produced AAS by Nassau Grouper in an hour. We then derived the modeling framework presented in Equation (1).

(1) $$\begin{array}{c} V_{j,i}\sim \text{Poisson}\;\left(\mu_{j,i}\right)\\ \log\left(\mu_{j,i}\right)=\log\tau_{j,i}+T_{i}+D_{i}+{\mathrm{FP}}_{j,i}+h_{j,i}\\ \begin{array}{c} \left[\begin{array}{c} h_{1,i}\\ \begin{array}{c} h_{2,i}\\ h_{3,i}\\ \begin{array}{c} h_{4,i}\\ h_{5,i} \end{array} \end{array} \end{array}\right]\sim \text{MVNormal}\;\left(\left[\begin{array}{c} 0\\ \begin{array}{c} 0\\ 0\\ \begin{array}{c} 0\\ 0 \end{array} \end{array} \end{array}\right],S_{H}\right)\\ S_{H}=\left(\begin{array}{c} \sigma_{h1}\\ \begin{array}{c} 0\\ 0\\ \begin{array}{c} 0\\ 0 \end{array} \end{array} \end{array}\;\begin{array}{c} 0\\ \begin{array}{c} \sigma_{h2}\\ 0\\ \begin{array}{c} 0\\ 0 \end{array} \end{array} \end{array}\;\begin{array}{c} 0\\ \begin{array}{c} 0\\ \sigma_{h3}\\ \begin{array}{c} 0\\ 0 \end{array} \end{array} \end{array}\;\begin{array}{c} 0\\ \begin{array}{c} 0\\ 0\\ \begin{array}{c} \sigma_{h4}\\ 0 \end{array} \end{array} \end{array}\;\begin{array}{c} 0\\ \begin{array}{c} 0\\ 0\\ \begin{array}{c} 0\\ \sigma_{h5} \end{array} \end{array} \end{array}\right)R\left(\begin{array}{c} 1\\ \begin{array}{c} \rho_{2,1}\\ \rho_{3,1}\\ \begin{array}{c} \rho_{4,1}\\ \rho_{5,1} \end{array} \end{array} \end{array}\;\begin{array}{c} \rho_{1,2}\\ \begin{array}{c} 1\\ \rho_{3,2}\\ \begin{array}{c} \rho_{4,2}\\ \rho_{5,2} \end{array} \end{array} \end{array}\;\begin{array}{c} \rho_{1,3}\\ \begin{array}{c} \rho_{2,3}\\ 1\\ \begin{array}{c} \rho_{4,3}\\ \rho_{5,3} \end{array} \end{array} \end{array}\;\begin{array}{c} \rho_{1,4}\\ \begin{array}{c} \rho_{2,4}\\ \rho_{1,2}\\ \begin{array}{c} 1\\ \rho_{5,4} \end{array} \end{array} \end{array}\;\begin{array}{c} \rho_{1,5}\\ \begin{array}{c} \rho_{2,5}\\ \rho_{3,5}\\ \begin{array}{c} \rho_{4,5}\\ 1 \end{array} \end{array} \end{array}\right)\\ \begin{array}{c} T_{i}\sim \text{Normal}\left(0,1\right)\\ D_{i}\sim \text{Normal}\left(0,1\right)\\ \begin{array}{c} {\mathrm{FP}}_{j,i}\sim \text{Normal}\left(0,1\right)\\ \sigma_{d}\sim \text{Exponential}\left(1\right)\\ R\sim \text{LKJcorr}\left(1\right) \end{array} \end{array} \end{array} \end{array}$$

In the above model formulation, the number of AAS detected at a given hydrophone and hour is a function of the log form of the minutes observed per hour (defined as 0 < 𝜏_*j,i*_ ≤ 1 h), time of day (24 categories reflecting hour of day that each hour of the study [*i*] belongs to; *T*_*i*_), day of the spawning season relative to the night of the first spawn (seven categories reflecting −4 to +2 days after first spawn [DAFS] that each hour of the study [*i*] belongs to; *D*_*i*_), proximity of the bulk of spawning fish relative to each hydrophone at each hour (categorical estimate with three levels; FP_*j,i*_), and a random effect of recording time interval (each hour of the study at each hydrophone; *h*_*j,i*_). We define these *h*_*j,i*_ values to be multivariate normal within each time interval in order to account for the expected correlated nature of AAS detections between hydrophones related to the spatial nature of fish behaviors and AAS at the spawning site. Within the covariance structure (**S**_*H*_), each hydrophone has a separate variance, while the covariance of hydrophones (**R**) is assumed to be fixed across hours. That is, we expect limited hydrophone detection ranges coupled with complex aggregating behaviors (e.g., dispersing from or coalescing to a specific location within the array as a function of time of day or ocean condition) would lead to correlations in AAS detections across hydrophones that would not be captured simply by the proximity of spawning fish to each hydrophone independently.

We specified weakly regularizing priors for all priors in our model formulation above and fit the model using Stan (Stan Development Team, 2022) via the ulam function in the Statistical Rethinking R package (McElreath, 2020) using R version 4.3.3 (R Core Team, 2023). We ran three chains concurrently with 1000 iterations per chain and evaluated convergence based on trace plots and Gelman‐Rubin scores (all parameters ~1, indicating efficient mixing among MCMC chains and model convergence). All data and model code used in our analysis are available at https://doi.org/10.5281/zenodo.15677765.

## RESULTS

Most hydrophones in the deployed array recorded for the duration of the study period. LS3 malfunctioned early in the recording period and did not gather sufficient data for analysis. In addition, ST4 abruptly ended its recording just after midnight on February 15, two days prior to the end of the study period. Despite these issues, we collected over 1000 h of acoustic data across the five functional hydrophones. Classifications from FADAR had an overall accuracy of 76.0%, and an associated Cohen's Kappa (*K*) estimate of 51.4. The software also produced an inflated false negative rate (FNR) of 46.8% and a low true positive rate (TPR) of 53.2%. However, an investigation into the classifier's performance for each hydrophone indicated particularly poor performance within ST2 and ST4. Upon viewing the spectrograms of these hydrophones, we found a consistent floor noise between 0 and 50 Hz which impacted the spectrogram images and thus FADAR's ability to detect present AAS (signaled by its markedly high FNR at ST2 and ST4; Table 2). Removing ST2 and ST4 samples from the cumulative subset improved FADAR's evaluation metrics considerably. Summed samples from LS1, LS5, and ST6 generated a high accuracy of 85.6%, high TPR of 73.5%, low FNR of 26.5%, and a *K* of 70.5. FADAR yielded a low average false positive rate (FPR) of 2.0% for all stations, with the well‐performing stations (LS1, LS5, and ST6) yielding a comparable average of 2.9%. The lower overall FPR across all summed stations was largely attributable to the floor noise issue with ST2 and ST4 which prevented FADAR from predicting many AAS, thus generating an FPR of 0 for both stations (Table 2).

**TABLE 2:** Parameters to measure accuracy and reliability of Nassau Grouper (*Epinephelus striatus*) aggregation‐associated sounds (AAS) classification by FADAR (Fish Acoustic Detection Algorithm Research) and human efforts, estimated using counts found in Table 1.

Station	Classifier	K	Acc	MCR	TPR	TNR	FPR	FNR
LS1	FADAR	0.696	0.880	0.120	0.634	0.995	0.005	0.366
ST2	FADAR	0.116	0.492	0.508	0.138	1.000	0.000	0.862
	HUMAN	0.683	0.838	0.162	0.829	0.850	0.150	0.171
ST4	FADAR	0.051	0.604	0.396	0.044	1.000	0.000	0.956
	HUMAN	0.668	0.841	0.159	0.812	0.906	0.094	0.188
LS5	FADAR	0.624	0.843	0.157	0.810	0.889	0.111	0.190
ST6	FADAR	0.681	0.847	0.153	0.672	0.983	0.017	0.328
All stations	FADAR	0.518	0.760	0.240	0.532	0.980	0.020	0.468
LS1, LS5, ST6	FADAR	0.708	0.856	0.144	0.735	0.971	0.029	0.265
ST2, ST4	HUMAN	0.677	0.840	0.160	0.819	0.876	0.124	0.181

Abbreviations: Acc, accuracy; FNR, false negative rate; FPR, false positive rate; *K*, Cohen's kappa; MCR, misclassification rate; TNR, true negative rate; TPR, true positive rate.

Because of FADAR's relatively poor performance on stations ST2 and ST4, we used manual visual classification as described in 2.4. Manual classification accuracy was comparable to that of FADAR within its well‐performing stations (LS1, LS5, ST6; Table 2) and performed better with higher TPR and lower FNR; however, it also yielded lower true negative rate (TNR) and higher FPR, with a marginally lower *K*. Manual classification efforts within stations ST2 and ST4 generated accuracy metrics that were highly comparable to each other, suggesting no bias in classified detections by the researcher within either sample.

### Spatiotemporal trends

**FIGURE 3:**
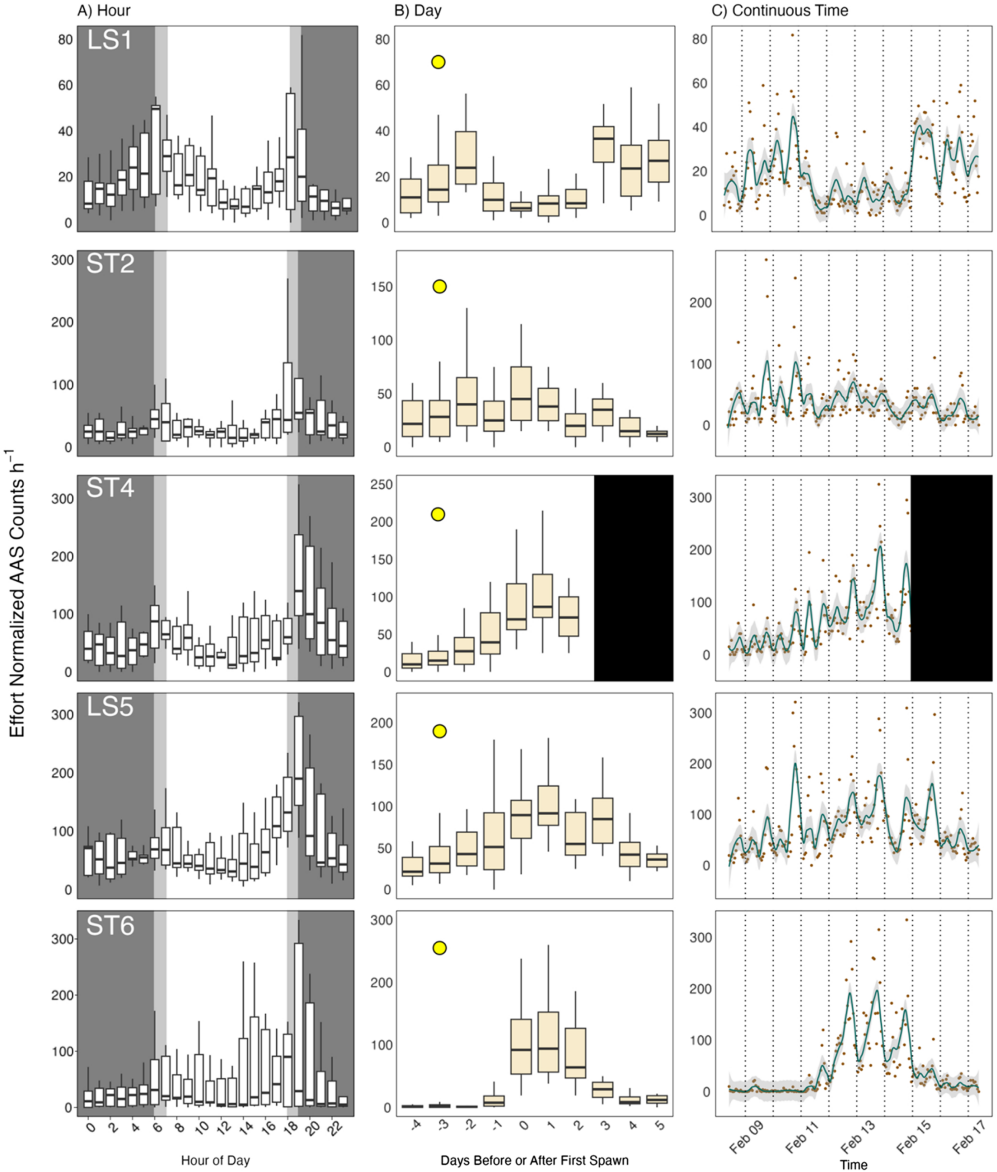
Temporal trends of effort normalized Nassau Grouper (*Epinephelus striatus*) aggregation‐associated sounds (AAS) detection rates across all hydrophones. Because ST4 abruptly halted recording at midnight of 3 days after the first night of spawning (DAFS), black rectangles cover 3–5 DAFS for ST4 in (b) and (c). (a) Box plots of AAS detection rates for all hours of day at each hydrophone. Dark gray rectangles signify hours at night while light gray rectangles signify hours in which the sun rose (06:00) and set (18:00). (b) Box plots of AAS detection rates per hour binned by day at each hydrophone. Yellow circles outlined black symbolize the full moon which occurred −3 DAFS. (c) AAS detection rates per hour for all continuous hours of observation. Orange points indicate effort normalized AAS counts per hour of day. A green spline fits to the orange points by the loess method with a span factor of 0.1. The dark gray shade around the fitted spline represents the 89% CI. Dotted black lines separate days. Note that the first night of spawning (DAFS 0) is February 12, 2020. For all box plots: the black bar of the box is the median value; the area of the box encapsulates the middle 50% of the data (i.e., the first and third quantiles of data); the whiskers extend to the data points nearest to 1.5 × the interquartile range from the first quantile (low end) and third quantile (high end); and outliers are omitted.

Regardless of distance from the FSA center, all hydrophones detected crepuscular patterns in Nassau Grouper AAS production rates (Figure 3a). Hours in which the sun rose or set had the highest average AAS detections per hour, specifically 06:00–08:00 and 18:00–20:00 (Figure 3a). All stations detected greater rates of AAS production in the evening hours compared to the morning, except for LS1, which detected an equivalent share of AAS in both periods. ST6 had the highest variability of AAS detection rates, as exemplified by low median rates and exceptionally high upper quartile ranges during most daylight hours (Figure 3a). Hydrophones nearest to the FSA center (ST4, LS5, and ST6) detected AAS more frequently during days in which individuals spawned (0 to +2 DAFS; Figure 3b). Call detections through time also displayed crepuscular patterns regardless of when fish spawned (Figure 3c). Both daylight and nighttime hours contained comparably low Nassau Grouper AAS detection rates. Hydrophone‐specific Nassau Grouper AAS detection rates also largely scaled with proximity to the bulk of Nassau Grouper (Figure 2). Apparent breakdowns in this relationship may be reflective of the complex social and temporal patterns in AAS production during the aggregation (e.g., increases in AAS during dawn/dusk periods; Figure 3a).

**FIGURE 4:**
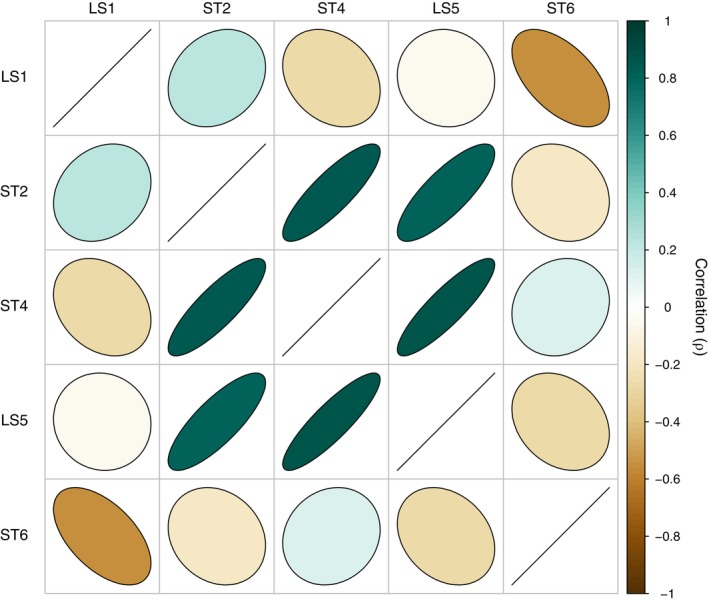
Correlation pairs plot of model‐predicted correlations between each hydrophone pair after accounting for all other model effects (i.e., time of day, day of spawning period, and proximity of the mass of fish). The slope and color of the ellipses symbolize the strength of correlation (e.g., strong positive correlations are narrow ellipses sloped positively and colored dark green). Straight, positively sloped lines occupy comparisons of identical pairs (e.g., LS1‐LS1).

**FIGURE 5:**
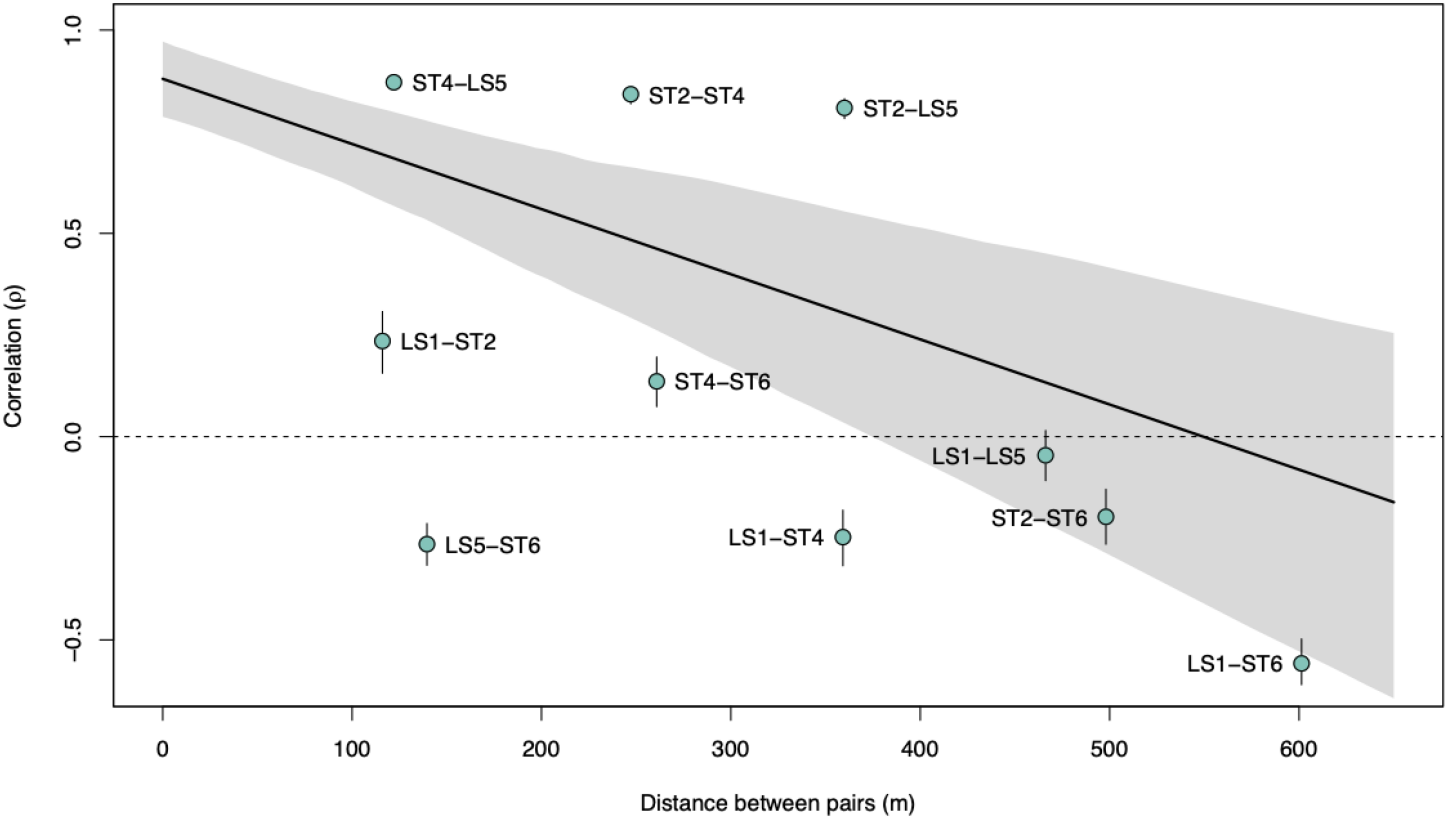
Linear regression of correlation coefficients against distance between hydrophone pairs in meters. Light green points symbolize the correlation coefficient of all hydrophone pairs at their distance of separation, with corresponding labels of the pair adjacent to each point. Whiskers extending from each point represent 25% and 75% posterior quantiles around the median estimated correlation. The 95% CI in the regression is shaded in light gray. A dashed black line extends from *y* = 0 for legibility. The “geosphere” package (Hijmans, 2024; version 1.5‐20) in R estimated distances between pairs of hydrophones from their coordinates.

Our model predicted higher correlations in AAS detections among neighboring hydrophone pairs than distant pairs through time (Figure 4). Predicted correlation (ρ) decreased with increasing distance between pairs, ranging from 0.87 to −0.56 at distances of 122.1 and 601.3 m between pairs, respectively (Figure 5). Correlation regressed over distance between pairs estimated ρ equaling zero at roughly 540 m of separation between hydrophones (Figure 5). The comparison of ST4–LS5 yielded the highest estimate of ρ (0.87, 122.6 m apart) while other comparisons of adjacent hydrophones yielded positive estimates. A high predicted correlation between ST2 and LS5 (ρ = 0.81) contrasted against their wide distance apart (360.0 m). Similarly, the model predicted LS5–ST6 to be poorly correlated (ρ = −0.27) despite their proximity (139.5 m).

### Model

**FIGURE 6:**
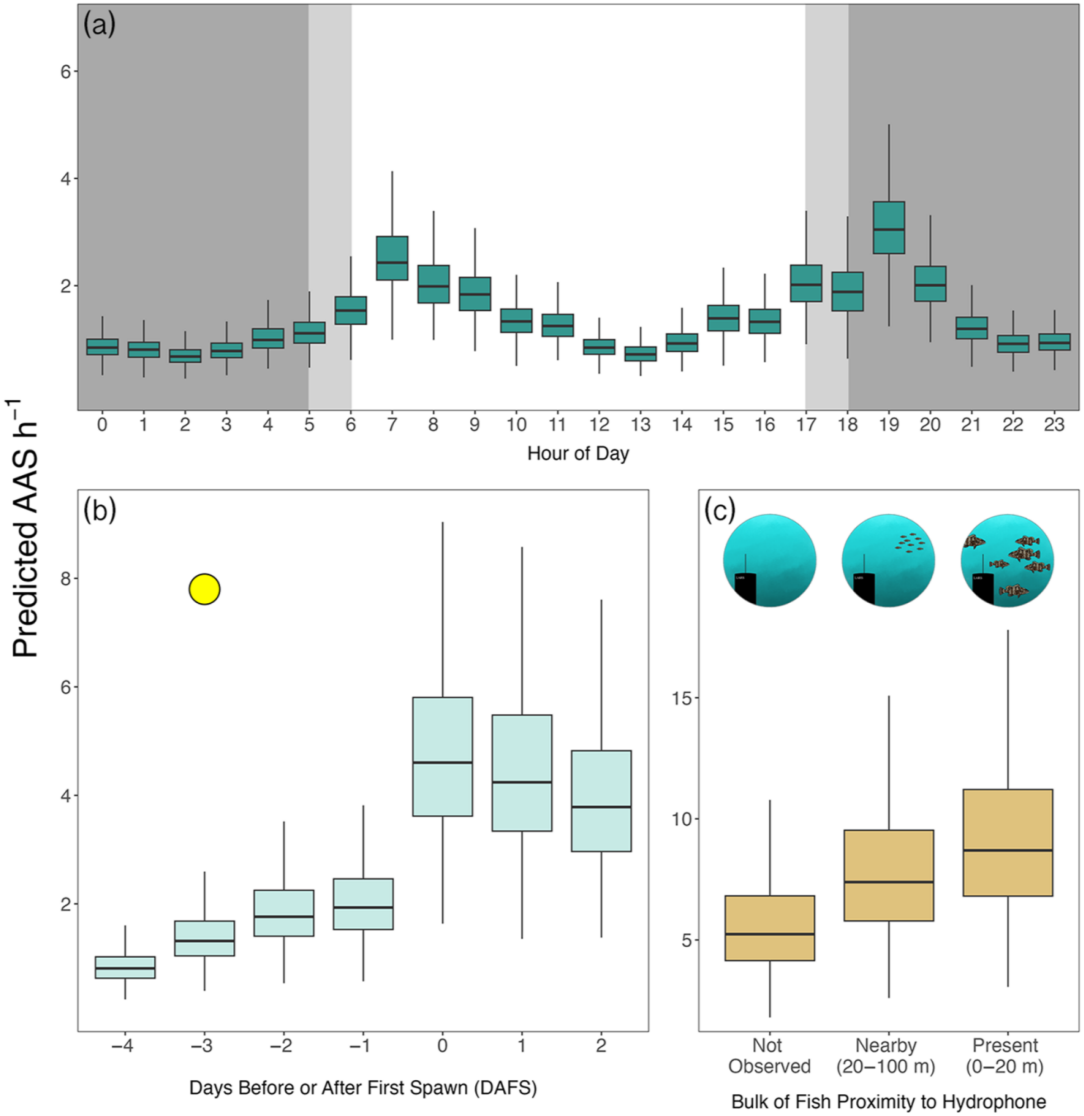
Box plots of posterior predictions of the parameters (a) *T*_*i*_, (b) *D*_*i*_, and (c) FP_*i*_ (see *Data analysis* for variable descriptions). (a) Posterior predictions of aggregation‐associated sounds (AAS) detection rates by *T*_*i*_. Dark gray panels signify nighttime hours while light gray panels represent the hours of sunrise (06:00) and sunset (18:00). (b) Posterior predictions of AAS detection rates by *D*_*i*_. A yellow circle outlined black signifies the full moon on −3 days after first spawn (DAFS). (c) Posterior predictions of AAS detection rates by FP_*i*_ . Above the boxplots are cartoons roughly representing each category of fish proximity to a hydrophone. For all box plots: the black bar of the box is the median value; the area of the box encapsulates the middle 50% of the data (i.e., the first and third quantiles of data); the whiskers extend to the data points nearest to 1.5 × the interquartile range from the first quantile (low end) and third quantile (high end); and outliers are omitted. Illustration credit: Cameron J. Van Horn.

Due to an anomalous error that caused ST4 to stop recording during the first hour of February 15, we only included data from all hydrophones (excluding malfunctional LS3) captured prior to February 15 (continuous observation inclusive from 10:00 February 8 to 0:00 February 15). Time of day, day of spawning period, and proximity of the mass of fish to each hydrophone were all strong predictors of AAS detection rates (Figure 6). Our model predicted Nassau Grouper AAS detection rates to fluctuate around crepuscular hours (Figure 6a, Appendix S1: Table S1). Model‐estimated Nassau Grouper AAS detection rates were higher during sunset (19:00) than sunrise (07:00). Crepuscular hours (defined as 06:00–08:59 and 18:00–20:59) had the highest estimated AAS detection rates. The model predicted hours leading up to and following crepuscular periods to gradually increase and decrease in AAS productivity, respectively. Nassau Groupers were least likely to produce AAS during the middle of the night (02:00) and midday (13:00).

Model‐estimated Nassau Grouper AAS detection rates steadily increased from the first day of observation and peaked during days in which spawning occurred (Figure 6b). The first day of observation (−4 DAFS) had the lowest predicted Nassau Grouper AAS detection rate. All days of observed spawning (0–2 DAFS, divers did not observe any spawning thereafter) shared high predicted AAS detection rates, contrasting the low rates for days prior to spawning. Fish proximity to the hydrophone strongly predicted hydrophone‐specific Nassau Grouper AAS detection rates (Figure 6c).

## DISCUSSION

Nearly all prior investigations into the temporal dynamics of spawning‐related AAS for Nassau Grouper and related tropical grouper species have relied on a single hydrophone deployment at or near the FSA. Given the potential for limited detection ranges relative to movements of fish at the spawning site (Schärer et al., 2012; Wilson et al., 2020), any temporal dynamics inferred from these studies at least partially reflect both temporal patterns in AAS and the movements of spawning fishes relative to the hydrophone. The goal of our study was to capture the spatial field of AAS across the spawning site per unit time, while simultaneously documenting dynamics in the location of spawning fish relative to each hydrophone in our array. By accounting for these spatial dynamics in our subsequent modeling efforts, we were able to quantify the influence of movement on AAS dynamics at the level of individual hydrophones. Moreover, we were able to separate the influence of spatial dynamics from the temporal dynamics in AAS (time of day, day of spawning period). The resulting estimates of temporal dynamics are thus uniquely “clean” estimates of temporal dynamics and rates of soniferous behaviors at the spawning site.

### FADAR

We employed the automatic classifier FADAR both to ease the burden of manually extracting Nassau Grouper AAS detections from over 1000 h of acoustic data and to evaluate the efficacy of the automatic classification technology for Nassau Grouper AAS. FADAR performed well in our dataset (with the exceptions due to hardware anomalies at ST2 and ST4) with high estimates of accuracy and Cohen's Kappa (Table 2). This technology and other classifiers of fish vocalizations thus hold great promise in the growing area of PAM research, especially within resource‐limited fisheries. Nonetheless, improvements to the FADAR tool would be of great benefit to the PAM community. For instance, incorporating the ability to specify the types of vocalizations produced by a given species would provide further insight into spatiotemporal complexities in intraspecies communication and behavior (and possibly highlight biasing in detection accuracy by a classifier). Also, because aggregating species produce hundreds of AAS in a given hour (Figure 2), finer temporal resolution attributed to detected vocalizations could improve estimates of AAS during spawning events at FSAs and better represent the true target soundscape.

The nature of studies employing PAM techniques often involves stationing recorders for remote data collection for long periods of time. FADAR and other machine learning automatic classifiers can save researchers post‐processing time on the order of months when compared to manual efforts to characterize embedded biological data in these datasets. It is nonetheless important that the performance of such automated classification tools be evaluated beyond the training environment under which they were built. FADAR's inconsistency in its own accuracy was most likely attributable to a possible hardware malfunction in ST2 and ST4 as the spectrograms of each hydrophone contained a very low frequency band (between 0 and 50 Hz). While hardware errors such as these have a minimal impact on manual, human classification efforts (as demonstrated in this study), they apparently can critically compromise automatic classification. We suggest that future studies take measures to ensure their instruments are free of any potential hardware issues that might impact how acoustic data are captured, such as testing their instruments in situ and inspecting spectrograms prior to long‐term stationing.

### Nassau Grouper calling trends

While the change in raw AAS detection rates across days of the spawning period varied widely by individual hydrophone (Figure 3c), the model‐estimated average daily AAS detection rates across the array (after accounting for spatial dynamics in fish behavior) showed a gradual increase following the full moon and an exponential increase once fish began spawning, with a peak on the 1st night of spawning (0 DAFS).

The relationship of the winter full moon and Nassau Grouper spawning behavior in the Caribbean has been reported in the literature as early as 1972 (Smith), and there is general consensus that the winter lunar cycle is a key environmental cue in migratory and aggregating behaviors (Bolden, 2000; Domeier & Colin, 1997; Sala et al., 2001). While the drivers behind individuals' directed migrations to the FSA are unclear, there is evidence that first‐time spawners follow more experienced fish, a behavior that may be mediated by sound production in older Nassau Groupers (Dahlgren et al., 2016; Rowell et al., 2015). The role of AAS in spatial organization at the FSA appears to be evident in the AAS production observed at LS1 and ST2. These hydrophones were the furthest to the Southeast (away from where the spawning ultimately occurred; Figure 1c) and recorded the highest AAS detection rates at the beginning and end of the spawning period, possibly when fish were entering/leaving the spawning grounds. Conversely, those hydrophones closest to where the FSA formed (Figure 1c) recorded the highest detection rates of AAS on spawning days (Figure 3b). Despite the fact that our detection data do not distinguish between Nassau Grouper AAS call types, we know from diver observations where the majority of Nassau Grouper aggregated throughout the spawning period, and specific days in which spawning occurred. Thus, we can reasonably infer the detected AAS after February 14 (the last day divers observed Nassau Grouper spawning) were non‐courtship related; we admit, however, that this inference should ultimately be tested in future work. Collectively, the hydrophones appear to capture the temporal pattern of arrival, staging, and ultimately coalescing to spawn, all of which are reflected in AAS variability across space and time.

Nassau Grouper produced the highest rates of AAS during nights of spawning (Figure 2). At hydrophones nearest to the aggregation, AAS production peaked from 0 to +2 DAFS, the same time frame in which divers observed spawning behavior (e.g., release of gametes into the water column). Nassau Grouper often produce the low‐frequency tonal call while exhibiting courtship behavior, which can include body coloration shifts (Archer et al., 2012; Schärer et al., 2012). Tonal AAS are hypothesized to signal a readiness to mate among individuals and to cue spawning synchrony for the greater population (i.e., FSA formation; Schärer et al., 2012). At the Little Cayman west end spawning site, peaks in tonal AAS occur as thousands of individuals concentrate into a narrow band along a short section of the shelf break. In previous studies, researchers were unable to partition the rise in spawning‐associated sound production due to the increased concentration of individuals versus increases in per capita AAS production. By accounting for the spatial distribution of fish at the spawning site across time in our model, we were able to approximately separate spatial effects of AAS detection rates from fish density/proximity effects on AAS detection rates, and in so doing demonstrate that both factors mediate the detection rates of AAS at a given hydrophone (Figure 6).

Nassau Grouper exhibited increased AAS production rates during crepuscular periods, regardless of the day of spawning or where on the spawning grounds they were (near or far from the FSA center). All hydrophone stations across the recording period recorded greater rates of AAS detection during dawn and dusk hours than all other times of day, with dusk typically containing the highest AAS density (Figure 3c). The increase in average AAS detection rates during hours leading up to sunset likely reflects individuals signaling readiness to spawn. Throughout their range, Nassau Groupers spawn near sunset, presumably because this timing reduces egg predation (Colin, 1992) and/or predation on spawning adults (e.g., Caribbean Reef Sharks *Carcharhinus perezi* at the study site, often observed attacking Nassau Grouper during gamete release). Overnight, simultaneous cross‐shelf wind‐driven currents help transport eggs onshore to nursery habitat (Shenker et al., 1993). AAS production near dusk may be an evolutionary response to motivate spawning that yields relatively high larval survival. However, increases in AAS in crepuscular periods hundreds of meters from the FSA center both during and outside nights of spawning suggest drivers behind AAS production extend beyond the immediate point and time of gamete release (Figure 2). Nassau Grouper may be calling upon arrival to the FSA as part of courtship behavior that signals readiness to mate. We observed that courtship behavior among individuals is protracted, occurring over multiple days and well before evenings of spawning—in such a scenario, mate selection at spawning may result from many previous nights of pairwise interactions, particularly during evening periods when individuals take on spawning colorations (Archer et al., 2012) that presumably reflect both fitness and readiness to spawn. Divers observed both behavioral (e.g., female herding; Whaylen et al., 2004) and coloration courtship signals (increasing proportions of bicolored individuals) prior to the day of first observed spawning, suggesting AAS production may extend from this anticipatory courtship behavior. Alternatively, AAS production could increase during crepuscular periods simply because these time periods are when Nassau Grouper are most active (regardless of spawning season). Blincow et al. (2020) found Nassau Groupers were more likely to increase vertical swimming activity in dawn and dusk hours and linked this behavior to possible hunting strategies. Nassau Groupers are known to hunt in light‐limited conditions as it benefits their ambush‐style predation (Carter et al., 1994). Though there is no present evidence that links AAS to hunting behavior, Nassau Groupers do produce a pulse AAS (described as akin to a heartbeat; Rowell et al., 2018) during agonistic displays of defense for a mate. The novelty of this AAS and its ties to aggression suggest sound production may be used to defend territories, or more generally as nonreproductive intraspecies communication, though this is speculative.

### Spatial variability

The location of hydrophones on the spawning grounds had a strong effect on the temporal variability in AAS detections. Model‐derived correlations among hydrophone datasets indicate the potential for the acoustic picture of an FSA to fully invert (highest AAS detection rates outside nights of peak spawning) if the distance between a hydrophone and the FSA exceeds only several hundred meters. While the time of day and spawning behavior by Nassau Grouper drove AAS production, the distance of the bulk of Nassau Grouper to the hydrophone strongly influenced AAS detection rates (Figure 2), suggesting variability in fish movement around an FSA can severely impact the interpretation of temporal acoustic trends. Our results indicate a clear need for future studies to either verify the proximities of fish near the recording hydrophone or alternatively implement a hydrophone array that captures spatiotemporal variability (within and between seasons) across the spawning grounds. This is particularly true if researchers intend to compare AAS production across spawning seasons or between spawning grounds, or approximate spawner abundance (relative or absolute) from detected AAS rates. Without accounting for the spatial effects inherent to AAS detection rates, the resulting findings are likely to be severely compromised.

Perhaps unsurprisingly, we found a strong decay in AAS detection rate correlations between hydrophones as a function of distance. Previous studies have suggested that the detection range of Nassau Grouper AAS at a particular hydrophone is roughly 200 m (Schärer et al., 2012; Wilson et al., 2020). Our findings generally support this range. Moreover, at a distance of 350 m, correlations between our hydrophone detections became consistently negative, a reflection of strong, small‐scale spatial patterns in AAS production on the spawning grounds. The similar positive correlations between LS5 and ST2, stationed over 300 m apart, and LS5 and ST6, separated by less than 150 m, are surprising (Figure 5). We believe that this complexity in positive correlations between hydrophones (after accounting for the covariates of spawn timing, time of day, and fish proximity) stems from the dynamics of aggregation formation and dispersal. Spawning, vocalizing fish exhibit complex behaviors and movement patterns across a large area, often coalescing and contracting during aggregation. It is likely that, in particular, the correlation between LS5 and ST2 reflects the fact that these hydrophone positions were typically at the outskirts of the location of where the mass of fish was present most of the period, and thus somewhat synchronized to the ingress and egress of spawning fish (Figure 2). From a monitoring perspective, the interactive effects of limited detection ranges and relatively fine‐scale acoustic behaviors mean that hydrophone placements are a critically important part of efforts to capture spawning dynamics and spawner abundance. To generalize our findings to future FSA studies of Nassau Grouper, we suggest researchers do preliminary scoping work to identify the extent of the FSA, and then field an array of hydrophones sufficient to capture this extent at approximately 200‐m intervals. Note that for some Nassau Grouper FSAs, a linear array may not be sufficient for this task (i.e., Nemeth et al., 2023).

FSAs are typically thought of as fixed, immutable locations that afford opportunities for population assessment that otherwise might be cost‐prohibitive or impossible given low densities of some aggregating species when they are on their home reefs. However, mounting evidence suggests that the spatial nature of aggregations is dynamic at scales ranging from 100 to 1000 s of meters or more, depending on species (Aguilar‐Perera, 2006; Caiger et al., 2020; Colin, 1992; Nemeth et al., 2020, 2023; Rowell et al., 2015). The Nassau Grouper FSA we targeted is no exception; during our study, we observed individuals spawning several hundred meters north of the traditional FSA location (based on previous years of FSA monitoring; Whaylen et al., 2007; Figure 1c). Because this shift in FSA location was larger than the apparent range of any given hydrophone, we captured trends in AAS detection rates on ST2 (the operational hydrophone nearest the traditional FSA site) that were not representative of the true trends in AAS detection rates across the spawning period—naively, such a finding in the absence of diver observations might be interpreted as evidence of spawning failure. More generally, AAS production patterns coupled with a weaker correlation among hydrophones nearer to the historical FSA compared to hydrophones nearer to the observed FSA suggest reliance on past estimates of FSA locations can introduce greater uncertainty in recorded data.

The diver and GoPro estimated the abundance of Nassau Grouper within 20–100 m of a given hydrophone had a clear effect on AAS detection rates for that hydrophone. In fact, regardless of the time of day or day of the spawning season, if an aggregation of Nassau Grouper is within 20 m of a hydrophone (present), AAS detection rates can be three times as high as when fish are not visibly present near a hydrophone (not observed; Figure 6c). This finding provides strong evidence that the movements of fish around the spawning grounds, on the scale of hours to days, can have dramatic impacts on hydrophone‐specific AAS detection rates that are unrelated to the tendencies of individual fish to call more or less frequently as a function of time. Why might this matter from a monitoring perspective? Even when researchers deploy an array of hydrophones to address spatial variance in calling rates, if unobserved fish movements at the spawning site are synchronized to temporal patterns of interest (e.g., the FSA moves in close proximity to a specific hydrophone each evening), spatial movements may erroneously be interpreted as temporal changes in AAS detection rates in subsequent modeling efforts. At a minimum, researchers should be aware of this potential confounding effect in future studies. Should time and support allow, it could be beneficial to pair some amount of visual monitoring (in situ or with imaging technologies) in order to help parse the relative effects of time and space in driving AAS detection rates.

The spectral structures of AAS and other biotic sounds are species‐specific, which enables researchers to distinguish between calls of several taxa. Particularly in tropical systems, FSAs can be composed of multiple soniferous species that have temporally overlapping spawning periods (Heyman & Kjerfve, 2008; Rhodes et al., 2014). Our study site is an example of such multispecies FSAs as it is the spawning ground for several other soniferous grouper species, including (Red Hind [*Epinephelus guttatus*], Yellowfin Grouper [*Mycteroperca venenosa*], and Black Grouper [*Mycteroperca bonaci*]). The AAS of these groupers share spectral qualities with that of Nassau Grouper, which, coupled with spatial overlap of aggregating behaviors, can contribute to a noisy soundscape that partially masks target AAS (Locascio & Burton, 2016; Wilson et al., 2020). Sonic interference can occur from anthropogenic sources as well. As we observed in our data, motorized vessels or other mechanical sources create loud, long‐lasting sounds at low frequencies that overlap with AAS of several fish species and can thus prevent detection (Webb et al., 2008). As with the potential for spatial movement patterns to generate spurious trends in AAS detection rates, biogenic or anthropogenic interference may also alter patterns in AAS detection rates.

The presumptive spatiotemporal predictability of FSAs and use of AAS by aggregating species has resulted in the broad adoption of PAM as an efficient, cost‐effective tool to collect data on aggregating populations. The fact that AAS detection rates generally scale with population density prompted several recent studies to investigate how accurately such rates can be used to infer fish stock size (Caiger et al., 2020; Looby et al., 2022; Rowell et al., 2012, 2017; Sanchez et al., 2017; Schärer et al., 2012). Despite these efforts, challenges persist. By using an array of hydrophones and monitoring the location of aggregating fishes in relation to these hydrophones across time, we were able to partition the competing effects of time, space, and behaviors on AAS detection rates. Understanding each of these effects and their collective influence on AAS detections at a given hydrophone is a necessary step in the effort to estimate population size from AAS detection rates. We thus suggest that any study focused on inferring ecological behaviors and distributions of a soniferous species should attempt to mitigate the effects of spatial heterogeneity in AAS detections when capturing and ultimately describing trends in AAS detection rates at FSAs. Otherwise, substantial error from imprecise methods may misrepresent a species' ecology and reduce the efficacy of subsequent management actions informed by such studies.

## CONFLICT OF INTEREST STATEMENT

The authors declare no conflicts of interest.

## Supporting information


Appendix S1:

## Data Availability

Data and code (Van Horn, 2025) are available in Zenodo at https://doi.org/10.5281/zenodo.15677765.

## References

[eap70081-bib-0001] Aalbers, S. A. , and C. A. Sepulveda . 2012. “The Utility of a Long‐Term Acoustic Recording System for Detecting White Seabass *Atractoscion nobilis* Spawning Sounds.” Journal of Fish Biology 81(6): 1859–1870. 10.1111/j.1095-8649.2012.03399.x. 23130687

[eap70081-bib-0002] Aguilar‐Perera, A. 2006. “Disappearance of a Nassau Grouper Spawning Aggregation Off the Southern Mexican Caribbean Coast.” Marine Ecology Progress Series 327(December): 289–296. 10.3354/meps327289.

[eap70081-bib-0003] Archer, S. K. , S. A. Heppell , B. X. Semmens , C. V. Pattengill‐Semmens , P. G. Bush , C. M. McCoy , and B. C. Johnson . 2012. “Patterns of Color Phase Indicate Spawn Timing at a Nassau Grouper *Epinephelus striatus* Spawning Aggregation.” Current Zoology 58(1): 73–83. 10.1093/czoolo/58.1.73.

[eap70081-bib-0004] Blincow, K. M. , P. G. Bush , S. A. Heppell , C. M. McCoy , B. C. Johnson , C. V. Pattengill‐Semmens , S. S. Heppell , et al. 2020. “Spatial Ecology of Nassau Grouper at Home Reef Sites: Using Acoustic Telemetry to Track a Large, Long‐Lived Epinephelid across Multiple Years (2005‐2008).” Marine Ecology Progress Series 655(November): 199–214. 10.3354/meps13516.

[eap70081-bib-0005] Bolden, S. K. 2000. “Long‐Distance Movement of a Nassau Grouper (*Epinephelus striatus*) to a Spawning Aggregation in the Central Bahamas.” Fishery Bulletin 98: 642–645.

[eap70081-bib-0006] Bush, P. G. , E. D. Lane , G. C. Ebanks‐Petrie , K. Luke , B. Johnson , C. McCoy , J. Bothwell , and E. Parsons . 2006. “The Nassau Grouper Spawning Aggregation Fishery of the Cayman Islands – An Historical and Management Perspective.” Gulf and Caribbean Fisheries Institute 57: 515–524.

[eap70081-bib-0007] Caiger, P. E. , M. J. Dean , A. I. DeAngelis , L. T. Hatch , A. N. Rice , J. A. Stanley , C. Tholke , D. R. Zemeckis , and S. M. Van Parijs . 2020. “A Decade of Monitoring Atlantic Cod *Gadus morhua* Spawning Aggregations in Massachusetts Bay Using Passive Acoustics.” Marine Ecology Progress Series 635(February): 89–103. 10.3354/meps13219.

[eap70081-bib-0008] Carter, J. , G. J. Marrow , and V. Pryor . 1994. “Aspects of the Ecology and Reproduction of Nassau Grouper (*Epinephelus striatus*) Off the Coast of Belize, Central America.” Proceedings of the Gulf and Caribbean Fisheries Institute 43: 65–111.

[eap70081-bib-0009] Colin, P. L. 1992. “Reproduction of the Nassau Grouper, *Epinephelus striatus* (Pisces: Serranidae) and Its Relationship to Environmental Conditions.” Environmental Biology of Fishes 34(4): 357–377. 10.1007/BF00004740.

[eap70081-bib-0010] Dahlgren, C. P. , K. Buch , E. Rechisky , and M. A. Hixon . 2016. “Multiyear Tracking of Nassau Grouper Spawning Migrations.” Marine and Coastal Fisheries 8(1): 522–535. 10.1080/19425120.2016.1223233.

[eap70081-bib-0011] De Mitcheson, Y. S. , A. Cornish , M. Domeier , P. L. Colin , M. Russell , and K. C. Lindeman . 2008. “A Global Baseline for Spawning Aggregations of Reef Fishes.” Conservation Biology 22(5): 1233–1244. 10.1111/j.1523-1739.2008.01020.x. 18717693

[eap70081-bib-0013] Domeier, M. L. , and P. L. Colin . 1997. “Tropical Reef Fish Spawning Aggregations: Defined and Reviewed.” Bulletin of Marine Science 60: 698–726.

[eap70081-bib-0014] Emslie, M. J. , A. J. Cheal , M. A. MacNeil , I. R. Miller , and H. P. A. Sweatman . 2018. “Reef Fish Communities Are Spooked by Scuba Surveys and May Take Hours to Recover.” PeerJ 6(May): e4886. 10.7717/peerj.4886. 29844998 PMC5971101

[eap70081-bib-0015] FAO . 2020. The State of World Fisheries and Aquaculture 2020. Sustainability in Action. Rome: FAO. 10.4060/ca9229en.

[eap70081-bib-0016] Fish, M. P. , and W. H. Mowbray . 1970. Sounds of Western North Atlantic Fishes: A Reference File of Biological Underwater Sounds. Baltimore, MD: The Johns Hopkins Press.

[eap70081-bib-0017] Gottesman, B. L. , J. C. Olson , S. Yang , O. Acevedo‐Charry , D. Francomano , F. A. Martinez , R. S. Appeldoorn , D. M. Mason , E. Weil , and B. C. Pijanowski . 2021. “What Does Resilience Sound Like? Coral Reef and Dry Forest Acoustic Communities Respond Differently to Hurricane Maria.” Ecological Indicators 126(July): 107635. 10.1016/j.ecolind.2021.107635.

[eap70081-bib-0019] Heppell, S. A. , B. X. Semmens , C. V. Pattengill‐Semmens , P. G. Bush , B. C. Johnson , C. M. McCoy , C. Paris , J. Gibb , and S. S. Heppell . 2009. “Tracking Potential Larval Dispersal Patterns from Nassau Grouper Aggregation Sites: Evidence for Local Retention and the ‘Importance of Place’.”

[eap70081-bib-0018] Heppell, S. A. , B. X. Semmens , S. K. Archer , C. V. Pattengill‐Semmens , P. G. Bush , C. M. McCoy , S. S. Heppell , and B. C. Johnson . 2012. “Documenting Recovery of a Spawning Aggregation through Size Frequency Analysis from Underwater Laser Calipers Measurements.” Biological Conservation 155(October): 119–127. 10.1016/j.biocon.2012.06.002.

[eap70081-bib-0020] Heyman, W. D. , and B. Kjerfve . 2008. “Characterization of Transient Multi‐Species Reef Fish Spawning Aggregations at Gladden Spit, Belize.” Bulletin of Marine Science 83(3): 531–551.

[eap70081-bib-0021] Hijmans, R. 2024. “geosphere: Spherical Trigonometry. R Package Version 1.5‐20.” https://github.com/rspatial/geosphere.

[eap70081-bib-0022] Ibrahim, A. K. , H. Zhuang , L. M. Chérubin , M. T. Schärer‐Umpierre , and N. Erdol . 2018. “Automatic Classification of Grouper Species by Their Sounds Using Deep Neural Networks.” The Journal of the Acoustical Society of America 144(3): EL196–EL202. 10.1121/1.5054911. 30424627

[eap70081-bib-0023] Ibrahim, A. K. , H. Zhuang , M. T. Schärer‐Umpierre , C. Woodward , N. Erdol , and L. M. Chérubin . 2024. “Fish Acoustic Detection Algorithm Research: A Deep Learning App for Caribbean Grouper Calls Detection and Call Types Classification.” Frontiers in Marine Science 11: 1378159. 10.3389/fmars.2024.1378159.

[eap70081-bib-0072] Kobara, S. , and W. D. Heyman . 2008. “Geomorphometric Patterns of Nassau Grouper (Epinephelus striatus) Spawning Aggregation Sites in the Cayman Islands.” Marine Geodesy 31(4), 231–245. 10.1080/01490410802466397.

[eap70081-bib-0024] Laiolo, P. 2010. “The Emerging Significance of Bioacoustics in Animal Species Conservation.” Biological Conservation 143(7): 1635–1645. 10.1016/j.biocon.2010.03.025.

[eap70081-bib-0025] Lindseth, A. , and P. Lobel . 2018. “Underwater Soundscape Monitoring and Fish Bioacoustics: A Review.” Fishes 3(3): 36. 10.3390/fishes3030036.

[eap70081-bib-0026] Lobel, P. S. 1992. “Sounds Produced by Spawning Fishes.” Environmental Biology of Fishes 33: 351–358.

[eap70081-bib-0027] Locascio, J. V. , and M. L. Burton . 2016. “A Passive Acoustic Survey of Fish Sound Production at Riley's Hump within Tortugas South Ecological Reserve; Implications Regarding Spawning and Habitat Use.” Fishery Bulletin 114(1): 103–116. 10.7755/FB.114.1.9.

[eap70081-bib-0028] Looby, A. , K. Cox , S. Bravo , R. Rountree , F. Juanes , L. K. Reynolds , and C. W. Martin . 2022. “A Quantitative Inventory of Global Soniferous Fish Diversity.” Reviews in Fish Biology and Fisheries 32(2): 581–595. 10.1007/s11160-022-09702-1.

[eap70081-bib-0029] Luczkovich, J. J. , D. A. Mann , and R. A. Rountree . 2008. “Passive Acoustics as a Tool in Fisheries Science.” Transactions of the American Fisheries Society 137(2): 533–541. 10.1577/T06-258.1.

[eap70081-bib-0030] Lyon, R. P. , D. B. Eggleston , D. R. Bohnenstiehl , C. A. Layman , S. W. Ricci , and J. E. Allgeier . 2019. “Fish Community Structure, Habitat Complexity, and Soundscape Characteristics of Patch Reefs in a Tropical, Back‐Reef System.” Marine Ecology Progress Series 609(January): 33–48. 10.3354/meps12829.

[eap70081-bib-0031] Mann, D. A. , and T. M. Grothues . 2009. “Short‐Term Upwelling Events Modulate Fish Sound Production at a Mid‐Atlantic Ocean Observatory.” Marine Ecology Progress Series 375(January): 65–71. 10.3354/meps07720.

[eap70081-bib-0033] Marques, T. A. , L. Thomas , S. W. Martin , D. K. Mellinger , J. A. Ward , D. J. Moretti , D. Harris , and P. L. Tyack . 2013. “Estimating Animal Population Density Using Passive Acoustics.” Biological Reviews 88(2): 287–309. 10.1111/brv.12001. 23190144 PMC3743169

[eap70081-bib-0034] McElreath, R. 2020. “rethinking: Statistical Rethinking Book Package.” R Package Version 2.01.

[eap70081-bib-0035] McHugh, M. L. 2012. “Interrater Reliability: The Kappa Statistic.” Biochemia Medica 22(3): 276–282. 10.11613/BM.2012.031. 23092060 PMC3900052

[eap70081-bib-0036] Milich, L. 1999. “Resource Mismanagement Versus Sustainable Livelihoods: The Collapse of the Newfoundland Cod Fishery.” Society & Natural Resources 12(7): 625–642. 10.1080/089419299279353.

[eap70081-bib-0037] Monczak, A. , Y. Ji , J. Soueidan , and E. W. Montie . 2019. “Automatic Detection, Classification, and Quantification of Sciaenid Fish Calls in an Estuarine Soundscape in the Southeast United States.” PLoS One 14(1): e0209914. 10.1371/journal.pone.0209914. 30650120 PMC6334970

[eap70081-bib-0038] Moulton, J. M. 1958. “The Acoustical Behavior of Some Fishes in the Bimini Area.” The Biological Bulletin 114(3): 357–374. 10.2307/1538991.

[eap70081-bib-0039] Munger, J. E. , D. P. Herrera , S. M. Haver , L. Waterhouse , M. F. McKenna , R. P. Dziak , J. Gedamke , S. A. Heppell , and J. H. Haxel . 2022. “Machine Learning Analysis Reveals Relationship between Pomacentrid Calls and Environmental Cues.” Marine Ecology Progress Series 681(January): 197–210. 10.3354/meps13912.

[eap70081-bib-0040] Nemeth, R. S. 2012. “Ecosystem Aspects of Species That Aggregate to Spawn.” In Reef Fish Spawning Aggregations: Biology, Research and Management. Fish & Fisheries Series 35., edited by Y. Sadovy de Mitcheson and P. Colin . Dordrecht: Springer. 10.1007/978-94-007-1980-4_2.

[eap70081-bib-0042] Nemeth, R. S. , E. Kadison , J. Jossart , M. Shivji , B. M. Wetherbee , and J. K. Matley . 2023. “Acoustic Telemtry Provides Insights for Improving Conservation and Management at a Spawning Aggregation Site of the Endangered Nassau Grouper (*Epinephelus striatus*).” Frontiers in Marine Science 10(March): 1–19. 10.3389/fmars.2023.1154689.

[eap70081-bib-0041] Nemeth, R. S. , E. Kadison , N. J. Brown Peterson , and J. Blondeau . 2020. “Reproductive Biology and Behavior Associated with a Spawning Aggregation of the Yellowfin Grouper *Mycteroperca venenosa* in the US Virgin Islands.” Bulletin of Marine Science 96(1): 31–56. 10.5343/bms.2019.0028.

[eap70081-bib-0043] R Core Team . 2023. R: A Language and Environment for Statistical Computing. Vienna: R Foundation for Statistical Computing. https://www.R-project.org.

[eap70081-bib-0044] Rhodes, K. L. , R. S. Nemeth , E. Kadison , and E. Joseph . 2014. “Spatial, Temporal, and Environmental Dynamics of a Multi‐Species Epinephelid Spawning Aggregation in Pohnpei, Micronesia.” Coral Reefs 33(June): 765–775. 10.1007/s00338-014-1172-z.

[eap70081-bib-0045] Rountree, R. A. , R. G. Gilmore , C. A. Goudey , A. D. Hawkins , J. J. Luczkovich , and D. A. Mann . 2006. “Listening to Fish: Applications of Passive Acoustics to Fisheries Science.” Fisheries 31(9): 433–446. 10.1577/1548-8446(2006)31[433:LTF]2.0.CO;2.

[eap70081-bib-0046] Rowell, T. J. , D. A. Demer , O. Aburto‐Oropeza , J. J. Cota‐Nieto , J. R. Hyde , and B. E. Erisman . 2017. “Estimating Fish Abundance at Spawning Aggregations from Courtship Sound Levels.” Scientific Reports 7(1): 3340. 10.1038/s41598-017-03383-8. 28611365 PMC5469787

[eap70081-bib-0048] Rowell, T. J. , M. T. Schärer , and R. S. Appeldoorn . 2018. “Description of a New Sound Produced by Nassau Grouper at Spawning Aggregation Sites.” Gulf and Caribbean Research 29: GCFI22–GCFI26. 10.18785/gcr.2901.12.

[eap70081-bib-0049] Rowell, T. J. , M. T. Schärer , R. S. Appeldoorn , M. I. Nemeth , D. A. Mann , and J. A. Rivera . 2012. “Sound Production as an Indicator of Red Hind Density at a Spawning Aggregation.” Marine Ecology Progress Series 462(August): 241–250. 10.3354/meps09839.

[eap70081-bib-0047] Rowell, T. J. , R. S. Nemeth , M. T. Schärer , and R. S. Appeldoorn . 2015. “Fish Sound Production and Acoustic Telemetry Reveal Behaviors and Spatial Patterns Associated with Spawning Aggregations of Two Caribbean Groupers.” Marine Ecology Progress Series 518(January): 239–254. 10.3354/meps11060.

[eap70081-bib-0050] Sadovy, Y. , A. Aguilar‐Perera , and E. Sosa‐Cordero . 2018. *Epinephelus striatus*: The IUCN Red List of Threatened Species. 2018. Report no. e. T7862A46909843. Gland: International Union for Conservation of Nature and Natural Resources. 10.2305/IUCN.UK.2018-2.RLTS.T7862A46909843.en.

[eap70081-bib-0051] Sadovy, Y. , and A. Eklund . 1999. “Synopsis of Biological Data on the Nassau Grouper, *Epinephelus striatus* (Bloch, 1792), and the Jewfish, E. Itajara (Lichtenstein, 1822).” NOAA Technical Report NMFS 146. https://repository.library.noaa.gov/view/noaa/3090/noaa_3090_DS1.pdf.

[eap70081-bib-0052] Sala, E. , E. Ballesteros , and R. M. Starr . 2001. “Rapid Decline of Nassau Grouper Spawning Aggregations in Belize: Fishery Management and Conservation Needs.” Fisheries 26(10): 8.

[eap70081-bib-0053] Sanchez, P. J. , R. S. Appeldoorn , M. T. Schärer‐Umpierre , and J. V. Locascio . 2017. “Patterns of Courtship Acoustics and Geophysical Features at Spawning Sites of Black Grouper (*Mycteroperca bonaci*).” Fishery Bulletin 115(2): 186–195. 10.7755/FB.115.2.5.

[eap70081-bib-0054] Schärer, M. T. , T. J. Rowell , M. I. Nemeth , and R. S. Appeldoorn . 2012. “Sound Production Associated with Reproductive Behavior of Nassau Grouper *Epinephelus striatus* at Spawning Aggregations.” Endangered Species Research 19(1): 29–38. 10.3354/esr00457.

[eap70081-bib-0055] Semmens, B. X. , K. E. Luke , P. G. Bush , C. V. Pattengill‐Semmens , B. Johnson , C. McCoy , and S. Heppell . 2007. “Investigating the Reproductive Migration and Spatial Ecology of Nassau Grouper (*Epinephelus striatus*) on Little Cayman Island Using Acoustic Tags – An Overview.” Gulf and Caribbean Fisheries Institute 58: 191–198.

[eap70081-bib-0056] Shenker, J. M. , E. D. Maddox , E. Wishinski , A. Pearl , S. R. Thorrold , and N. Smith . 1993. “Onshore Transport of Settlement‐Stage Nassau Grouper *Epinephelus striatus* and Other Fishes in Exuma Sound, Bahamas.” Marine Ecology Progress Series 98: 31–43. 10.3354/meps098031.

[eap70081-bib-0057] Smith, C. L. 1972. “A Spawning Aggregation of Nassau Grouper, *Epinephelus striatus* (Bloch).” Transactions of the American Fisheries Society 101(2): 257–261. 10.1577/1548-8659(1972)101<257:ASAONG>2.0.CO;2.

[eap70081-bib-0058] Stan Development Team . 2022. “Stan Modeling Language Users Guide and Reference Manual, Version 2.31.” https://mc-stan.org.

[eap70081-bib-0059] Starr, R. M. , E. Sala , E. Ballesteros , and M. Zabala . 2007. “Spatial Dynamics of the Nassau Grouper *Epinephelus striatus* in a Caribbean Atoll.” Marine Ecology Progress Series 343(August): 239–249. 10.3354/meps06897.

[eap70081-bib-0060] Stock, B. C. , S. A. Heppell , L. Waterhouse , I. C. Dove , C. V. Pattengill‐Semmens , C. M. McCoy , P. G. Bush , G. Ebanks‐Petrie , and B. X. Semmens . 2021. “Pulse Recruitment and Recovery of Cayman Islands Nassau Grouper (*Epinephelus striatus*) Spawning Aggregations Revealed by In Situ Length‐Frequency Data.” ICES Journal of Marine Science 78(1): 277–292. 10.1093/icesjms/fsaa221.

[eap70081-bib-0061] Stowell, D. , T. Petrusková , M. Šálek , and P. Linhart . 2019. “Automatic Acoustic Identification of Individuals in Multiple Species: Improving Identification across Recording Conditions.” Journal of the Royal Society Interface 16(153): 20180940. 10.1098/rsif.2018.0940. 30966953 PMC6505557

[eap70081-bib-0062] Teh, L. C. L. , and U. R. Sumaila . 2013. “Contribution of Marine Fisheries to Worldwide Employment: Global Marine Fisheries Employment.” Fish and Fisheries 14(1): 77–88. 10.1111/j.1467-2979.2011.00450.x.

[eap70081-bib-0063] Van Horn, C. 2025. “Cameron Van Horn/Nassau‐Grouper‐AAS‐Proximity‐Project: EA_article (Publication).” Zenodo. 10.5281/zenodo.15677766.

[eap70081-bib-0064] Waterhouse, L. , S. A. Heppell , C. V. Pattengill‐Semmens , C. McCoy , P. Bush , B. C. Johnson , and B. X. Semmens . 2020. “Recovery of Critically Endangered Nassau Grouper (*Epinephelus striatus*) in the Cayman Islands Following Targeted Conservation Actions.” Proceedings of the National Academy of Sciences of the United States of America 117(3): 1587–1595. 10.1073/pnas.1917132117. 31907312 PMC6983384

[eap70081-bib-0065] Webb, J. F. , R. R. Fay , and A. N. Popper , eds. 2008. Fish Bioacoustics. Springer Handbook of Auditory Research 32. New York: Springer.

[eap70081-bib-0067] Whaylen, L. , C. V. Pattengill‐Semmens , B. X. Semmens , P. G. Bush , and M. R. Boardman . 2004. “Observations of a Nassau Grouper, *Epinephelus striatus*, Spawning Aggregation Site in Little Cayman, Cayman Islands, Including Multi‐Species Spawning Information.” Environmental Biology of Fishes 70(3): 305–313. 10.1023/B:EBFI.0000033341.57920.a8.

[eap70081-bib-0066] Whaylen, L. , P. Bush , B. Johnson , K. E. Luke , C. McCoy , B. X. Semmens , and M. Boardman . 2007. “Aggregation Dynamics and Lessons Learned from Five Years of Monitoring at a Nassau Grouper (*Epinephelus striatus*) Spawning Aggregation in Little Cayman, Cayman Islands, BWI.” Gulf and Caribbean Fisheries Institute 59: 413–422.

[eap70081-bib-0068] Whitmarsh, S. K. , P. G. Fairweather , and C. Huveneers . 2017. “What Is Big BRUVver Up To? Methods and Uses of Baited Underwater Video.” Reviews in Fish Biology and Fisheries 27(1): 53–73. 10.1007/s11160-016-9450-1.

[eap70081-bib-0069] Wilson, K. C. , B. X. Semmens , C. V. Pattengill‐Semmens , C. McCoy , and A. Širović . 2020. “Potential for Grouper Acoustic Competition and Partitioning at a Multispecies Spawning Site off Little Cayman, Cayman Islands.” Marine Ecology Progress Series 634(January): 127–146. 10.3354/meps13181.

[eap70081-bib-0070] Winemiller, K. O. , and K. A. Rose . 1992. “Patterns of Life‐History Diversification in North American Fishes: Implications for Population Regulation.” Canadian Journal of Fisheries and Aquatic Sciences 49(10): 2196–2218. 10.1139/f92-242.

[eap70081-bib-0071] Worm, B. , and T. A. Branch . 2012. “The Future of Fish.” Trends in Ecology & Evolution 27(11): 594–599. 10.1016/j.tree.2012.07.005. 22877983

