# Supplementary material for: Hydrophone placement yields high variability in detection of Epinephelus striatus calls at a spawning site
Source: Ecol Appl. 2025 Aug 6;35(5):e70081. doi: 10.1002/eap.70081 (PMC12326518; doi:10.1002/eap.70081)
Supplement: Supplementary file 1 — Appendix S1: [file EAP-35-e70081-s001.pdf]

Ecological Applications
Article

Hydrophone placement yields high variability in detection of *Epinephelus striatus* calls at a spawning site

Cameron J. Van Horn, Alli C. Candelmo, Scott A. Heppell, Croy R.M. McCoy, Christy V. Pattengill-Semmens, Lynn Waterhouse, Laurent M. Cherubin, J. Christopher Taylor, William Michaels, James Locascio, Ali K. Ibrahim, Brice X. Semmens

APPENDIX S1

Table S1: Posterior of parameters estimated by Bayesian hierarchical model. ‘CorrPair’ parameters signify estimated correlations between hydrophone pairs (denoted by their station number within the brackets). Bolded lines indicate local maximums of posterior means. Independent samples exceeded 100 for each parameter estimate, with all R-hat values (indicating Markov Chain convergence to the target distribution) near 1.00. AAS = aggregation-associated sound; SD = standard deviation; DAFS = days after first spawn.

Parameter	Posterior Mean (Log AAS/hr)	Posterior SD (Log AAS/hr)	95% Confidence Interval	
			Lower	Upper
CorrPair[1,1]	1.000	0.000	1.000	1.000
CorrPair[1,2]	0.223	0.102	0.017	0.411
CorrPair[1,4]	-0.260	0.093	-0.432	-0.070
CorrPair[1,5]	-0.059	0.082	-0.223	0.089
CorrPair[1,6]	-0.550	0.084	-0.699	-0.370
CorrPair[2,1]	0.223	0.102	0.017	0.411
CorrPair[2,2]	1.000	0.000	1.000	1.000
CorrPair[2,4]	0.837	0.032	0.767	0.892
CorrPair[2,5]	0.799	0.036	0.722	0.858
CorrPair[2,6]	-0.189	0.099	-0.381	0.005
CorrPair[4,1]	-0.260	0.093	-0.432	-0.070
CorrPair[4,2]	0.837	0.032	0.767	0.892
CorrPair[4,4]	1.000	0.000	1.000	1.000
CorrPair[4,5]	0.869	0.027	0.811	0.918
CorrPair[4,6]	0.133	0.089	-0.049	0.297
CorrPair[5,1]	-0.059	0.082	-0.223	0.089
CorrPair[5,2]	0.799	0.036	0.722	0.858
CorrPair[5,4]	0.869	0.027	0.811	0.918
CorrPair[5,5]	1.000	0.000	1.000	1.000
CorrPair[5,6]	-0.262	0.078	-0.420	-0.111
CorrPair[6,1]	-0.550	0.084	-0.699	-0.370
CorrPair[6,2]	-0.189	0.099	-0.381	0.005
CorrPair[6,4]	0.133	0.089	-0.049	0.297
CorrPair[6,5]	-0.262	0.078	-0.420	-0.111
CorrPair[6,6]	1	0	1	1
sigma_d	1.152	0.049	1.058	1.242
00:00	-0.110	0.275	-0.664	0.436
01:00	-0.161	0.259	-0.683	0.351
02:00	-0.322	0.271	-0.857	0.183
03:00	-0.177	0.269	-0.712	0.342
04:00	0.057	0.262	-0.437	0.588
05:00	0.177	0.254	-0.320	0.684
**06:00**	**0.489**	**0.250**	**0.024**	**0.986**
**07:00**	**0.963**	**0.241**	**0.488**	**1.410**
**08:00**	**0.758**	**0.259**	**0.235**	**1.254**
**09:00**	**0.663**	**0.255**	**0.156**	**1.165**
10:00	0.362	0.252	-0.138	0.838
11:00	0.294	0.246	-0.182	0.763
12:00	-0.112	0.259	-0.624	0.381
13:00	-0.272	0.270	-0.796	0.267
14:00	-0.005	0.260	-0.520	0.512
15:00	0.392	0.253	-0.132	0.895
16:00	0.347	0.258	-0.148	0.870
**17:00**	**0.773**	**0.270**	**0.246**	**1.312**
**18:00**	**0.688**	**0.248**	**0.190**	**1.182**
**19:00**	**1.169**	**0.249**	**0.690**	**1.653**
**20:00**	**0.752**	**0.249**	**0.257**	**1.258**
21:00	0.232	0.253	-0.248	0.709
22:00	-0.026	0.261	-0.538	0.484
23:00	-0.005	0.262	-0.545	0.506
- 4 DAFS	-0.193	0.361	-0.932	0.504
- 3 DAFS	0.286	0.343	-0.372	0.952
- 2 DAFS	0.596	0.349	-0.090	1.292
- 1 DAFS	0.674	0.354	-0.058	1.330
**0 DAFS**	**1.531**	**0.346**	**0.864**	**2.195**
**1 DAFS**	**1.458**	**0.348**	**0.775**	**2.140**
**2 DAFS**	**1.348**	**0.344**	**0.649**	**1.992**
Not Observed	1.591	0.368	0.895	2.335
Nearby	1.927	0.365	1.226	2.666
**Present**	**2.095**	**0.368**	**1.393**	**2.827**

Figure S1: Map of Little Cayman marked with important places of interest. (a) Bathymetry map of Little Cayman. A gradient of blue hues marks depths binned by ranges of 10 meters and black land above sea level. A black box signifies the area mapped in (b). (b) Insert of Fish Spawning Aggregation (FSA) range at Little Cayman's west end. Dives conducted during the study period occurred only in this range. Divers used landmarks, moorings, and hydrophones displayed in this map to describe fish presence and movement. The blue hues of depth used in (a) extend to (b). LS = LS1 hydrophone; ST = SoundTrap Model 300HF hydrophone.

Figure S2: Time series of low frequency (0-600 Hz) interference events at each hydrophone on the order of minutes with coupled histograms on the order of hours. In the time series, gray bars signify minutes containing significant low frequency interference in the acoustic data that prompted their removal from their respective hydrophone's dataset. The adjacent histograms depict frequencies of minutes removed within each hour of a day. LS = LS1 hydrophone; ST = SoundTrap Model 300HF hydrophone.

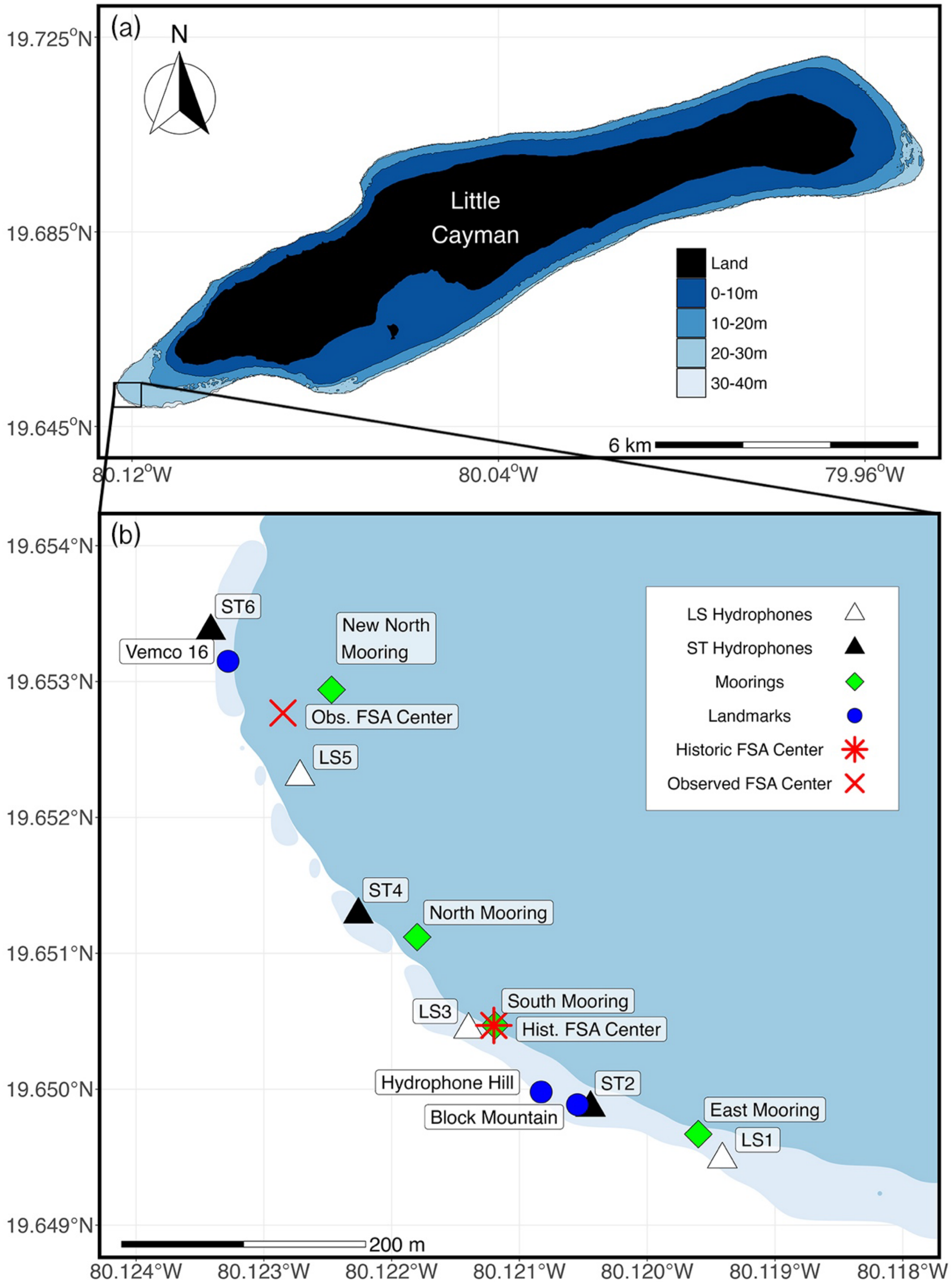

(a)
N
19.725°N
19.685°N
19.645°N
Little
Cayman
Land
0-10m
10-20m
20-30m
30-40m
6 km
80.12°W
80.04°W
79.96°W
(b)
19.654°N
19.653°N
19.652°N
19.651°N
19.650°N
19.649°N
ST6
Vemco 16
New North
Mooring
Obs. FSA Center
LS5
ST4
North Mooring
South Mooring
LS3
Hist. FSA Center
Hydrophone Hill
ST2
Block Mountain
East Mooring
LS1
LS Hydrophones
ST Hydrophones
Moorings
Landmarks
Historic FSA Center
Observed FSA Center
200 m
80.124°W
80.123°W
80.122°W
80.121°W
80.120°W
80.119°W
80.118°W

Figure S1

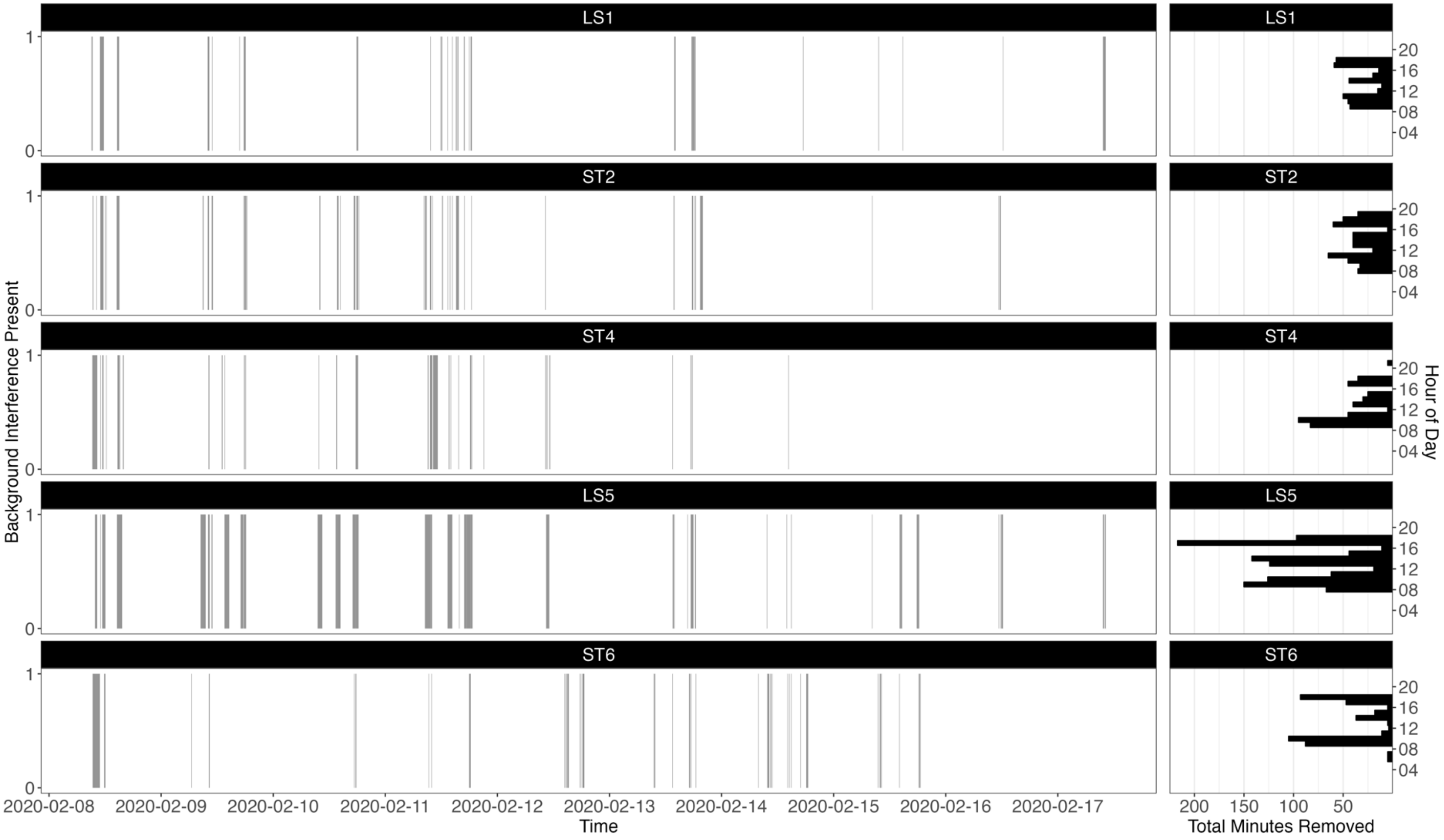
LS1
ST2
ST4
LS5
ST6
Background Interference Present
1
0
2020-02-08
2020-02-09
2020-02-10
2020-02-11
2020-02-12
2020-02-13
2020-02-14
2020-02-15
2020-02-16
2020-02-17
Time
20
16
12
08
04
Hour of Day
200
150
100
50
Total Minutes Removed

Figure S2
